# Identification of intestinal and fecal microbial biomarkers using a porcine social stress model

**DOI:** 10.3389/fmicb.2023.1197371

**Published:** 2023-11-09

**Authors:** Tuan Q. Nguyen, Marina Martínez-Álvaro, Joana Lima, Marc D. Auffret, Kenneth M. D. Rutherford, Geoff Simm, Richard J. Dewhurst, Eric T. Baima, Rainer Roehe

**Affiliations:** ^1^Scotland’s Rural College, Edinburgh, United Kingdom; ^2^Department of Animal Breeding, Faculty of Animal Science and Veterinary Medicine, Nong Lam University – Ho Chi Minh City, Ho Chi Minh City, Vietnam; ^3^Global Academy of Agriculture and Food Security, Royal (Dick) School of Veterinary Studies, University of Edinburgh, Edinburgh, United Kingdom; ^4^Zoetis Inc., Parsippany-Troy Hills, NJ, United States

**Keywords:** porcine microbiota, stress, growth, feed conversion ratio, feed intake, diversity, pathogen resistance

## Abstract

Understanding the relationships between social stress and the gastrointestinal microbiota, and how they influence host health and performance is expected to have many scientific and commercial implementations in different species, including identification and improvement of challenges to animal welfare and health. In particular, the study of the stress impact on the gastrointestinal microbiota of pigs may be of interest as a model for human health. A porcine stress model based on repeated regrouping and reduced space allowance during the last 4 weeks of the finishing period was developed to identify stress-induced changes in the gut microbiome composition. The application of the porcine stress model resulted in a significant increase in salivary cortisol concentration over the course of the trial and decreased growth performance and appetite. The applied social stress resulted in 32 bacteria being either enriched (13) or depleted (19) in the intestine and feces. Fecal samples showed a greater number of microbial genera influenced by stress than caecum or colon samples. Our trial revealed that the opportunistic pathogens *Treponema* and *Clostridium* were enriched in colonic and fecal samples from stressed pigs. Additionally, genera such as *Streptococcus*, *Parabacteroides*, *Desulfovibrio*, *Terrisporobacter*, *Marvinbryantia*, and *Romboutsia* were found to be enriched in response to social stress. In contrast, the genera *Prevotella*, *Faecalibacterium*, *Butyricicoccus*, *Dialister*, *Alloprevotella*, *Megasphaera*, and *Mitsuokella* were depleted. These depleted bacteria are of great interest because they synthesize metabolites [e.g., short-chain fatty acids (SCFA), in particular, butyrate] showing beneficial health benefits due to inhibitory effects on pathogenic bacteria in different animal species. Of particular interest are *Dialister* and *Faecalibacterium*, as their depletion was identified in a human study to be associated with inferior quality of life and depression. We also revealed that some pigs were more susceptible to pathogens as indicated by large enrichments of opportunistic pathogens of *Clostridium, Treponema, Streptococcus* and *Campylobacter*. Generally, our results provide further evidence for the microbiota-gut-brain axis as indicated by an increase in cortisol concentration due to social stress regulated by the hypothalamic–pituitary–adrenal axis, and a change in microbiota composition, particularly of bacteria known to be associated with pathogenicity and mental health diseases.

## 1. Introduction

Stress can be defined as the organism’s biological response to a threat or a disturbance to its homeostatic state. An organism’s stress response involves multiple important biological systems, particularly the autonomic nervous and neuroendocrine systems. The first drives alterations in the cardiovascular and gastrointestinal systems and delivers short duration responses (Cannon, 1929; Kemeny, 2003; Ziegler, 2012). In contrast, the neuroendocrine system response provides slower but potentially longer-lasting responses to challenges. In mammals, the main neuroendocrine response to stress involves the hypothalamic-pituitary-adrenal (HPA) axis, which affects many aspects of biology including metabolism, reproduction, and development of immunocompetence. The main output of the HPA axis are the glucocorticosteroids cortisol or corticosterone (depending on species) which regulate carbohydrate, fat, and protein metabolism, boost energy supplies, increase blood pressure and sugar as well as modulate the anti-inflammatory response. When animals are faced with challenges that quickly resolve, the role of the HPA axis and other stress response systems is adaptive. However, when challenges persist or are severe over the long-term, repeated or continual activation of the axis can be highly demanding on animal resources and lead to maladaptive outcomes such as reductions in immunocompetence, reproductive ability, growth development, productivity and welfare, as reflected by findings in many domesticated species including cattle (West, 2003), chickens (Zaboli et al., 2019), and pigs (Rutherford et al., 2006).

In pig production systems, sources of stress can be categorized into environmental, animal handling/management, and social causes (Martínez-Miró et al., 2016). Pigs are susceptible to ambient temperature, particularly high temperature (Mutua et al., 2020) since they lack functional sweat glands and have relatively small lungs to effectively dissipate excess heat (D’Allaire et al., 1996; Patience et al., 2005). Farming operations involving animal handling (such as vaccination, snaring, blood sampling, ear tagging or tattooing) or transport (uploading, unloading, travel duration and vehicle design) are also stressful for pigs (Prunier et al., 2005; Goumon and Faucitano, 2017). Additional sources of stress include for example, high stocking density, which leads to restricted space allowance, reduced feeder space per animal, lower access to the feeder, and increased competition between the animals, resulting in more frequent fighting, and higher levels of aggression (Randolph et al., 1981; Andersen et al., 2004), and animal regrouping, which can cause changes in the social dynamics of the population, leading to fights and subsequent harassment of the defeated animals (D’Eath, 2002; Desire et al., 2015a; Foister et al., 2018).

Stress and intestinal microbiome appear to be closely linked. Bailey et al. (2011) found that mice exposed to social disruption exhibited an increased relative abundance of *Clostridium* and decreased relative abundance of *Bacteroidetes* in comparison to their non-stressed counterparts. Within the pig industry, weaning represents a highly stressful challenge because piglets suddenly experience new nutritional, physiological, and psychological challenges. Weaning-induced stress is reported to result in a decline of some benefit bacterial genera such as *Alloprevotella* and *Oscillibacter*, while some opportunistic pathogens such as *Campylobacter, Clostridium XlVa,* and *Clostridium XlVb* increased in colon (Li et al., 2018). In addition, studies over the last decade have provided important information on a bidirectional microbiome-gut-brain axis, in which the gut microbiota and their metabolites interact with the host’s brain over metabolic, immunological, endocrine and neural pathways (Cryan and O’Mahony, 2011; Grenham et al., 2011; Wang and Kasper, 2014; Martin et al., 2018; Valles-Colomer et al., 2019; Barandouzi et al., 2020). As suggested by Bohórquez et al. (2014) and Bohórquez and Liddle (2015), the gut microbiota and their metabolites communicate through the gut connectome (i.e., the complex neural network in the gut involving the gut glial, intrinsic neurons and enteroendocrine cells), playing an important role in gut enteric nerves development and enhancement of the host’s ability to sense and use nutrients. Additionally, Messaoudi et al. (2011) found that a probiotic based on *Lactobacillus helveticus* R0052 and *Bifidobacterium longum* R0175 contributed to reducing anxiety-like behaviors in rats and alleviated psychological distress in humans. Furthermore, when transplanting the fecal microbiome of depressed human patients to antibiotics-treated rats, Kelly et al. (2016) found that the rats exhibited anxiety-like behaviors and tryptophan metabolism disruption, and proposed the vital role of the intestinal microbiome on development of depression in humans.

Most studies on the bidirectional microbiome-gut-brain axis have been performed in mice, for example by using germ-free or specific-pathogen-free (regulated by antibiotics) animals, or by performing fecal microbiome transplants (Martin et al., 2018). Previous authors have investigated the shift of the intestinal microbiome due to weaning stress in pigs (Guevarra et al., 2018; Li et al., 2018), but overall, much less literature is available on the microbiome-gut-brain axis on livestock, particularly on growing pigs. The present study is the first to use a social stress model to investigate the effect of stress on the intestinal and fecal microbiota in finishing pigs. A combination of high stocking density, low feed space and regular regrouping was employed to ensure the reliable establishment of stress in pigs. Each of the above stressors has been found to increase stress levels in pigs, with many biological consequences such as abnormal maternal behaviors (Jarvis et al., 2006; Ison et al., 2010), tail biting and aggression behaviors (Turner et al., 2001; Cornale et al., 2015), cortisol and testosterone elevation (Rutherford et al., 2006; Coutellier et al., 2007; Escribano et al., 2015), and negative cognitive bias (Scollo et al., 2014).

Pigs have previously been shown to be a highly relevant model species for studying the effect of environmental factors (e.g., nutrition, milk delivery method, antibiotic treatment) on early-life microbiota establishment in humans (Roura et al., 2016). Pigs have an omnivorous nature, with diet, nutritional requirements, and size similar to humans (Heinritz et al., 2016). Regarding physical and physiological traits, the digestive systems of pigs and humans are very similar, including the transit rate of liquids and food in the gastrointestinal tract, and the digestive and absorptive processes (Graham and Åman, 1987; Miller and Ullrey, 1987). Humans and pigs are essential colon fermenters (Graham and Åman, 1987) of plant/fibrous dietary components. Considering the gut microbiota composition, *Firmicutes* and *Bacteroidetes* are the dominant phyla in humans and pigs (Heinritz et al., 2013). Due to its size and temperament, the pig provides an easier approach for sample collection, such as saliva, blood samples and cannulas in the gastrointestinal tract, than other species, e.g., mice. Enhanced by innovative techniques, such as genetically modified, germ-free, gnotobiotic and human-microbial associated pigs, the pig is emerging as a powerful, translational model of gastrointestinal microbiota studies (Rose et al., 2022). In contrast to mice, pigs and humans have the common HPA axis’s output of cortisol with a similar circadian rhythm in responding to stress (Ruis et al., 1997), resulting in similar effects on the immune system, gastrointestinal and neuroendocrine alterations (Gimsa et al., 2018). Therefore, these authors concluded that social stress in pigs can reflect partly the intensity of psychosocial stress in the human society. These findings suggest that application of the social-stress model in pigs provides a great opportunity to research the modulation effect of stress on the gut microbiota, as well as the potential use of microbiota as therapeutic or preventive tools to cope with the stress challenge in humans. The potential microbial biomarkers of social stress are expected to have also implementation for dietary intervention (e.g., development of probiotics) and animal breeding for pathogen resistant pigs.

The objectives of this study were (i) to demonstrate the effectiveness of a porcine stress model, (ii) to assess the effects of social stress on porcine performances and intestinal (caecum and colon) and fecal microbiota, (iii) to identify social stress microbial biomarkers in the intestinal and fecal samples of pigs with potential for broader applications in other species, and (iv) to identify potential differences of animals in susceptibility to intestinal pathogen growth.

## 2. Materials and methods

### 2.1. Ethics statement

The porcine stress trial was conducted at the Pig Research Centre of Scotland’s Rural College (SRUC). The experiment was approved by SRUC’s Animal Welfare and Ethical Approval Body and was conducted in accordance with the requirements of the UK Animals (Scientific Procedures) Act 1986.

### 2.2. Animals

**Figure 1:**
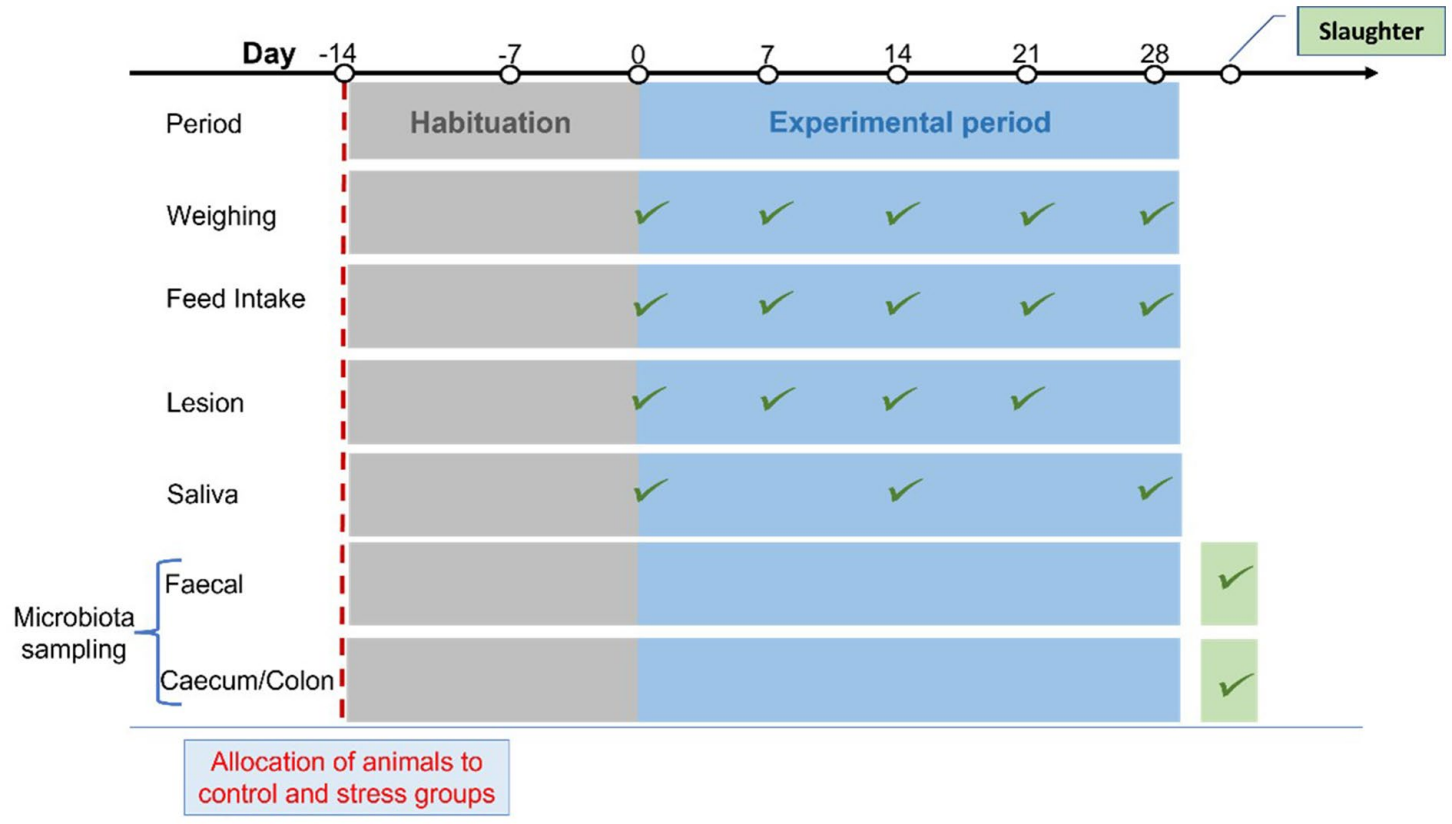
Schematic diagram of the experimental timeline, including habituation before the stress trial period presenting the recordings and samples used in this study.

The pigs included in the experiment were bred, raised, and tested at SRUC’s Pig Research Centre. They were obtained from 9 litters of crosses of Hampshire boars and crossbred Large White × Landrace sows. Litters were weaned at ~28 days of age and kept in mixed sex groups under normal farm conditions until study animals were selected for us at approximately 10 weeks of age. The 40 intact male pigs used in the study were selected based on good health and were randomly allocated to either the stress or control groups within the restriction to achieve groups being as far as possible balanced for body weight and litter of origin. Pigs from each litter were available in both experimental groups. At about 3.5 months of age, the pigs were moved into the experimental building and housed in 10 pens of 4 pigs per pen for a two-week habituation period before the start of the four-week experiment. During the adaptation period, the animals underwent a habituation regime designed to adapt them to the processes of weighing, fecal sampling, and saliva sampling; two pigs (one per group) were removed from the trial due to lameness. A total of 38 pigs entered the four-week trial with half of the pigs in the stressed and half in the control group. During the stress trial, the pigs were weighed weekly and feed intake was recorded at the pen level (Figure 1). Individual feed intake (DFI) was estimated by the methodology originally proposed by Lindemann and Kim (2007) and further validated by Lee et al. (2016).

The pigs were fed *ad libitum* a complete compound diet with a dry matter composition of 16.5% crude protein, 1% lysine, 0.29% methionine, 3.8% oil (acid ether extract), 4.25% ash, 16.7 neutral-detergent fiber, 38.7 starch and 5.3% crude fiber. The digestible energy content of the diet was 13.5 MJ/kg DM. Total weight gain during the stress trial was divided by the total days to obtain average daily gain (ADG, kg/day). Feed conversion ratio (FCR) was calculated as DFI divided by individual ADG.

### 2.3. Treatments

The stressors imposed on the treatment group were (i) the weekly regrouping of animals (Stress = regrouping was conducted according to a pre-planned schedule which aimed to maximize the degree of unfamiliarity between each successive batch of four pigs, i.e., to limit the degree to which pigs were mixed into groups with pigs they had encountered in a previous week, or who were original littermates; Control = no regrouping), (ii) low space allocation (Stress = ~1 m^2^ per pig; Control = ~2 m^2^ per pig), and (iii) limited feeder space (Stress = 30 cm per pen; Control = 60 cm per pen). Pigs in the stress group was ensured to be with new pen mates each week and had no repeated pen mate during the trial. Some repeated encounters of littermates were unavoidable due to the low number of litters and animals. However, the repeatedly mixed setting of littermates is expected to break down the effect of familiarity (littermate or not) in pig aggression which are often observed in a simple single mixing procedure (Giersing and Andersson, 1998).

### 2.4. Skin lesions score analysis

The number of new skin lesions (hereinafter referred to as total lesion score) was evaluated as a proxy indicator of the degree of aggression experienced by individual pig. The total number of lesions was counted for each pig on the day prior to each regrouping (i.e., prior to the first mix and then 6 days after subsequent mixing). All new (identified by redness, blood – new or old, or sebaceous fluid present) and freshly scabbed (where removal would see redness underneath or fresh bleeding) individual lesions were counted in three body areas, front, middle and rear on left and right side of the pig. The front area includes the whole head and ear to the shoulder blade and front leg. The middle area ranges from the shoulder to the back hip and includes the belly and spine; the rear area starts from the back hip including the back leg and tail and anogenital area. Lesions that are almost entirely healed (very faded, brown thin scabs which on rubbing show only unaffected/new skin underneath) were not counted (Turner et al., 2006).

### 2.5. Cortisol analysis

HPA activation as an indication of stress level was assessed by measurements of salivary cortisol. Saliva samples were collected using a cotton bud swab (MillPledgeVeterinary, UK) at the end of the habituation period as well as at the end of weeks 2 and 4 during the stress trial. Saliva samples were collected at 07:00, 10:00, 13:00, and 16:00 on each day of sampling. Determination of the cortisol concentration as the area under the curve (AUC) of the four measures per day of cortisol release was used as a summary measure of cumulative cortisol output on the assessed days.

The cortisol concentration in the saliva samples was determined in 96 wells plates following the protocol of a quantitative enzyme immunoassay kit obtained from ALPCO (Salem, NH, United States).

### 2.6. Collection of fecal and intestinal samples

Fecal samples were collected per rectum at the end of the trial. About 5 g of homogenized fecal samples were stored in 30 mL universal containers (Alphalabs, UK) filled with 4 mL RNALater (Sigma-Aldrich, UK), before being snap-frozen and stored at −80°C.

At the end of the trial, pigs were moved in whole groups from the experimental building to a nearby building where they were sedated and euthanized, prior to dissection and tissue collection. Slaughtering pigs at the same facility as they were tested avoided stress during transportation to the abattoir and stress prior to death was prevented using a sedative mixture (Ketamine, Azaperone, Medetomidine) injected intramuscularly prior to an overdose of Pentobarbital sodium (Euthatal) via injection to the heart. Following confirmation of death, intestinal luminal contents and mucosal cell wall samples were collected from the caecum and mid-colon. Intestinal luminal content samples were collected in universal 30 mL tubes and stored at −80 prior to analyzes.

### 2.7. DNA extraction, 16S rRNA gene sequencing, and identification of microbial taxonomy

Total DNA was extracted from intestinal content and feces samples following an adapted protocol of Yu and Morrison (2004) by combining chemical lysis and bead beating. The process continued with purification on columns using the QIASymphony with the Qiagen Midi kit. DNA was finally eluted in 400 μL of EB (Qiagen, UK) and an aliquot of 200 μL was directly stored at −20°C. The amount of DNA extracted was quantified by Qubit fluorometric quantitation for dsDNA (ThermoFisher, UK).

An adapted protocol based on the 16S Metagenomic Sequencing Library Preparation for the Illumina MiSeq System (Illumina, UK) was applied for total DNA extracted from feces, caecum, and mid-colon samples. The V4 region of the 16S rRNA sequences was amplified using primers 515F and 806R. From a broader project, two 16S libraries were composed of 95 and 93 amplicon samples purified on magnetic beads using the ProNex Chemistry (Promega, WI, United States) and quantified using Qubit assay prior to being pooled in two different tubes. In particular, 82 amplicon samples (38, 38, and 6 from caecal, colonic, and fecal samples, respectively) and 32 amplicon samples (fecal) in this current study were contributed into the two libraries, respectively. An aliquot of 10 ng/μl in 15 μL per library was sent to Edinburgh Genomics (Scotland, UK) for Illumina sequencing using MiSeq v2 250PE and providing a yield of at least 11 M + 11 M reads per run. The 16S rRNA amplicon sequences, and quality scores associated to each nucleotide in each read obtained from the Illumina procedure were analyzed with the pipeline QIIME2. The primers were removed by q2-cutadapt plugin available in QIIME2. The quality scores were accessed visually through interactive quality boxplots, resulting in a manual decision to trim forward and reverse reads at 153 and 157 bases, respectively. DADA2 denoising workflow was provided by QIIME2 for amplicon sequence variants identification (Callahan et al., 2016). For taxonomy classification, a pre-trained Naive Bayes classifier provided by QIIME2 was applied. The classifier (silva-132-99-515-806-nb-classifier.qza) was trained on SILVA database (release 132) for the V4 region bounded by the 515F/806R primer pair (as used in this current study). More details can be found in Lima et al. (2019). One table of hit counts was created for each taxonomic level (Domain, Phylum, Class, Order, Family and Genus). As a result, 146 genera from 61 families and 17 phyla were identified from 114 samples.

### 2.8. Statistical analyzes

#### 2.8.1. Taxonomic diversity

Alpha-diversity of each sample was assessed through richness (i.e., number of observed taxa) and adjusted Shannon index (Veech, 2017). Beta-diversity was explored using Bray–Curtis dissimilarity. A linear mixed model was fitted separately for each sampling location including control/stress treatment as fixed effect and litter as random effect to estimate the impact of the stress condition on alpha-diversity. For beta-diversity, a non-parametric permutational multivariate analysis of variance (PERMANOVA) with 999 Monte Carlo permutations was conducted (Warton et al., 2012) separately for each sampling site fitting treatment and litter as fixed effects. The analyzes were performed using lme4 (Bates et al., 2015) and vegan (Oksanen et al., 2020) R packages considering a *p*-value < 0.05 as statistically significant.

#### 2.8.2. Data filtering and cleaning.

The microbial datasets at the genera level contained 57% of zero values. The minimum prevalence threshold to discard non-informative microbial taxa (i.e., minimum % of animals that should contain the microbial genera), was determined using the Prevalence Interval for Microbiome Evaluation (PIME) workflow from the R package PIME (Roesch et al., 2020) separately for each sampling site. Briefly, PIME uses a machine-learning algorithm to find the optimal prevalence threshold that maximizes the discrimination between treatments (control and stress), evaluated through the out-of-bag error. Subsequently, genera with an average relative abundance lower than 0.001 were removed from the dataset, leaving 49, 54, and 57 genera identified in the caecum, colon, and feces samples, respectively. The remaining zero values were imputed by using the ANOVA-Like Differential Expression (ALDEx2) method from the R package ALDEx2 (Fernandes et al., 2013, 2014). ALDEx2 substitutes the zeros with the posterior probabilities of each taxon using Monte-Carlo sampling from a Dirichlet distribution. The proportional data was then transformed into components by centered log-ratio (*clr*) transformation to account for the compositional nature of microbiota datasets (Greenacre, 2018). The *clr* data then was used in further analyzes.

#### 2.8.3. Discriminant analysis

We used a sequential partial least squares discriminant analyzes (PLSDA)-based methodology to identify which microbes mostly lead to the discrimination between stressed and control treatment groups. Three different models were fitted, each within each sample type (i.e., caecum, colon, and feces). First, PLSDA analyzes were carried out using the number of latent components determined by ‘leave-one-out’ cross-validation (based on the minimum classification error), and taxa that obtained variable importance in projection (VIP) score lower than 0.8 were removed from the analyzes. In subsequent PLSDA analyzes, one latent component was consistently selected by cross-validation as the best option and the taxa that had a VIP < 0.8 were again removed. This procedure was continued until the PLSDA analysis started losing discrimination ability. The genera included in the final PLSDA models were identified as being the most important variables for the discrimination between the control and stress groups. These genera were then evaluated with Welch’s *t*-test to assess their enrichment in each treatment group using the ALDEx2 package (Fernandes et al., 2013, 2014). The identified biomarkers (predictors), together with the pig samples classified by control or stress treatments (response variables) were represented graphically in the PLSDA biplots (Oyedele and Lubbe, 2015; Rohart et al., 2017).

### 2.9. Identification of porcine pathogen resistance

**Figure 2:**
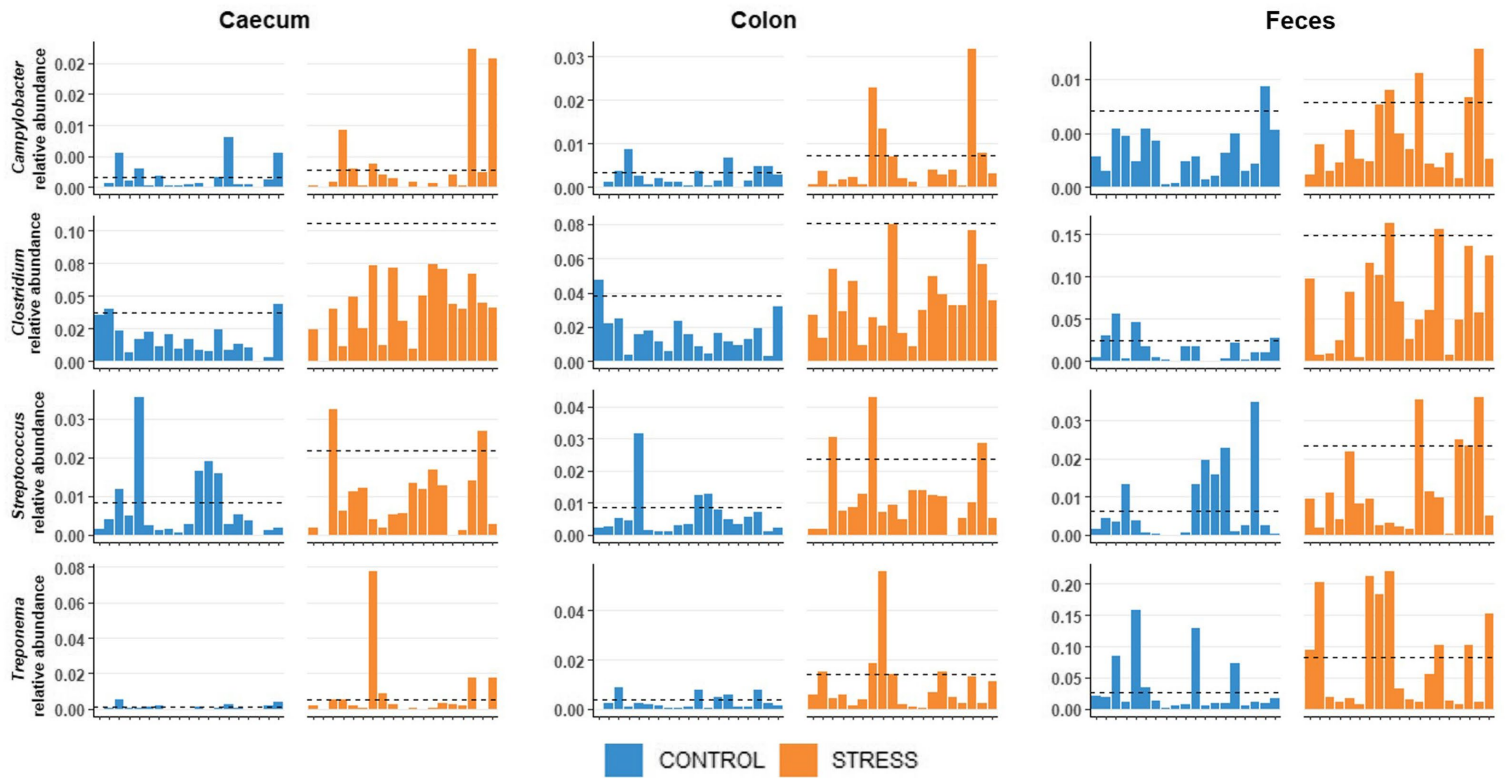
Distribution of relative abundances of *Clostridium, Treponema, Streptococcus*, and *Campylobacter* of the pigs in stress and control groups.

We observed that, independently of the treatment (stress/control), some hosts were more resistant (i.e., presented lower abundance) to specific pathogenic bacteria (*Clostridium, Treponema, Streptococcus* and *Campylobacter*, see Figure 2), which might influence the results on these specific bacteria obtained in the stress/control discrimination analysis (PLSDA and Welch’s test). The potential resistance may involve several mechanisms, such as, antimicrobial peptides, secretory immunoglobulin, mucosal immune system, pH levels, resource competition from commensal bacteria, resulting in reducing the growth of pathogens in the lumen of the intestine (Khan et al., 2021). We first divided the animals into resistant or susceptible groups. After exploratory analyzes, animals were considered susceptible to a specific pathogenic bacterium when the relative abundance of this bacterium was at least 2.5 times greater than the median relative abundance within their treatment group (for each of the sampling sites). The median was chosen to identify the animals with most extreme abundances, whereas the mean was identified to be unsuitable due to the high number of animals showing low abundances. The value of 2.5 times the median was determined by visual inspection of the extreme abundances of the pathogenic genera across animals. Considering a normal distribution, the value 2.5 times the mean, would represent a significance level of 0.006 using a one-side statistical test. The Bayesian regression model was fitted for each of the sampling site [using brms package with default settings (Bürkner, 2021)] with stress/control treatment together with susceptible/resistant groups as fixed effects while litter was fitted as random effect to explore the potential impact of stress on these pathogenic genera’s *clr* abundances.

## 3. Results

### 3.1. Skin lesions

**Figure 3:**
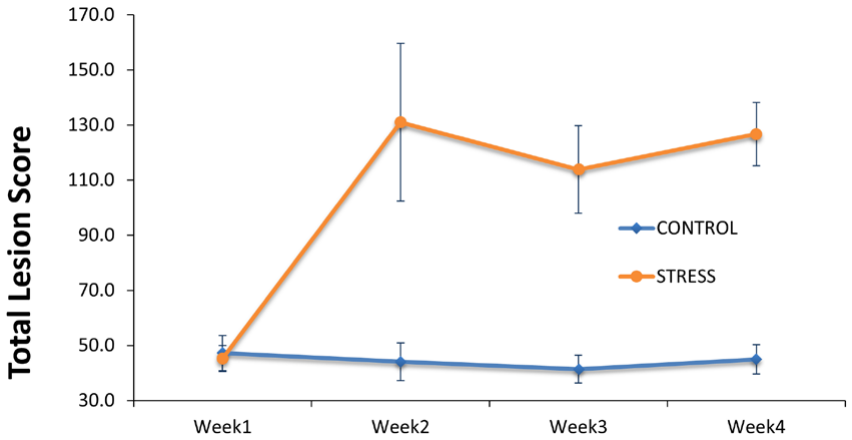
Comparison of lesion score between the stress and control groups. Skin lesions of the entire body were counted at the start of the trial before regrouping of animals between pens and during the trial at the start of Week 2 to Week 4 in which the animals of the stress group have been mixed between pens weekly.

To assess the consequences of any aggression occurring between animals after regrouping, individual total lesion score was counted and summarized in Figure 3 as average values for the stress and control groups before and during the stress trial. At the start of the trial and before the regrouping of animals, the average numbers of skin lesions were similar at 45.3 ± 4.7 and 47.3 ± 6.4 in animals allocated to the stress and control groups, respectively. After weekly regrouping of animals between pens, the number of skin lesions was three-fold larger in the stress group (131.1 ± 28.6) than in the control (44.1 ± 6.9) in week 2 and this difference was maintained until the end of the experiment. This indicates the substantial amount of fighting within the stress group. When considering the whole experimental period, the stress group showed a significantly higher total lesion score (59.7 ± 9.9) than the control group (*p* < 0.001) (Table 1).

**Table 1:** Least squared mean and the difference between stress and control groups in growth performance, cortisol concentration AUC, and total lesion score.

	Control		Stress		Difference	SE	*p*-value
Trait	Mean	SE	Mean	SE			
DFI (kg)	2.79	0.07	2.44	0.08	0.35	0.11	<0.001
ADG (kg)	1.33	0.03	1.11	0.03	0.21	0.04	<0.001
FCR (kg DFI/kg ADG)	2.11	0.05	2.23	0.05	−0.12	0.07	0.09
Cortisol concentration	192.49	16.55	299.62	16.55	−107.13	23.40	<0.001
Total lesion score	44.42	7.00	104.11	7.05	−59.69	9.93	<0.001

### 3.2. Salivary cortisol concentration

**Figure 4:**
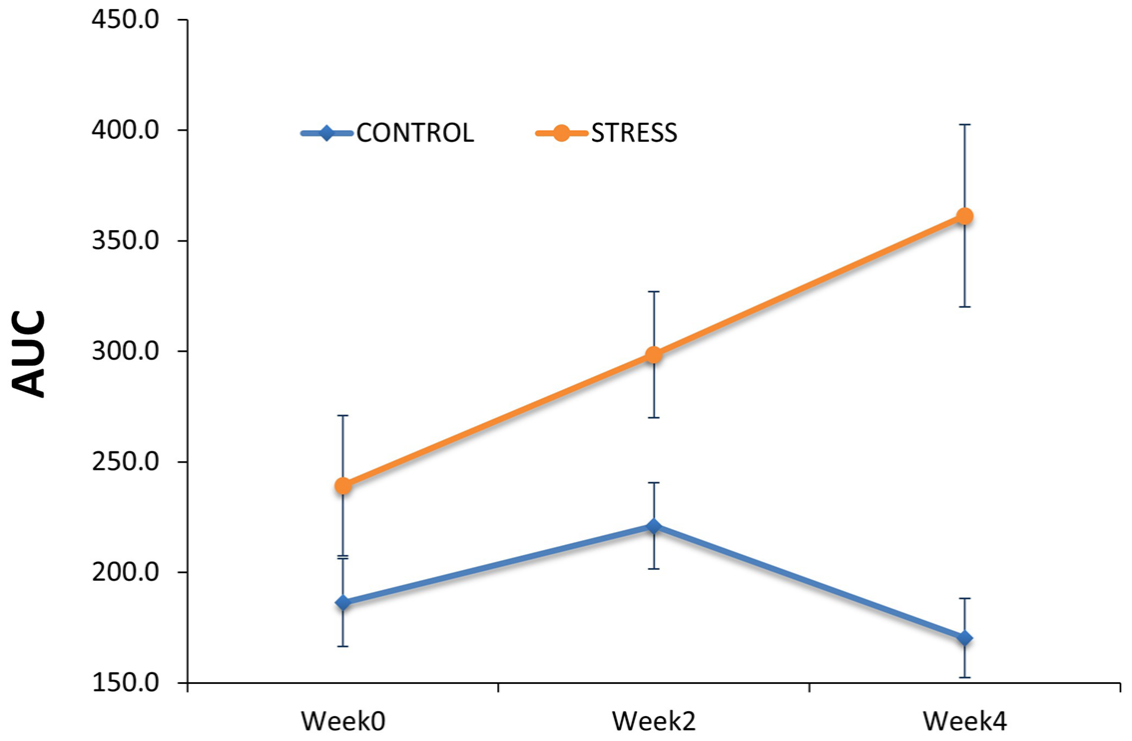
Comparison of salivary cortisol concentration between the stress and control groups. Salivary cortisol concentration was determined as the area under the curve (AUC) to capture the cumulative cortisol exposure per day within the stress and control groups before (Week0) and during the stress trial (Week2 and Week4).

The salivary cortisol concentration was compared between groups (Table 1). At the end of the habituation period (prior to the exposure to social stress, week 0), non-significant differences were observed in cortisol AUC level (AUC_Stress_ – AUC_Control_ = 53 ± 37.5, *p* = 0.16, Figure 4).

However, differences between groups gradually increased over the stress treatment period, with the stress group showing 77.4 ± 34.7 (*p* = 0.03) larger AUC cortisol concentration at week 2 and 191 ± 44.9 (*p* < 0.01) at week 4. These results confirm that the imposed stressors (i.e., weekly regrouping of pigs, higher stocking density and reduced feeder space) substantially affected the psycho-neuroendocrinological system of the animals.

### 3.3. Performance traits

Over the entire experiment, animals exposed to social stress had a significant reduction in daily feed intake and average daily gain of 0.35 kg of feed/day and 0.21 kg of weight/day, respectively, in comparison to the control group (Table 1).

The reduction in feed intake due to stress is likely to have contributed to the impairment of the growth rate. As expected, animals exposed to social stress were less efficient in converting feed into body weight gain (i.e., needed more feed per kg average daily gain indicated by a higher feed conversion ratio), although not significantly so (*p* = 0.09).

### 3.4. Microbiota profiles

**Figure 5:**
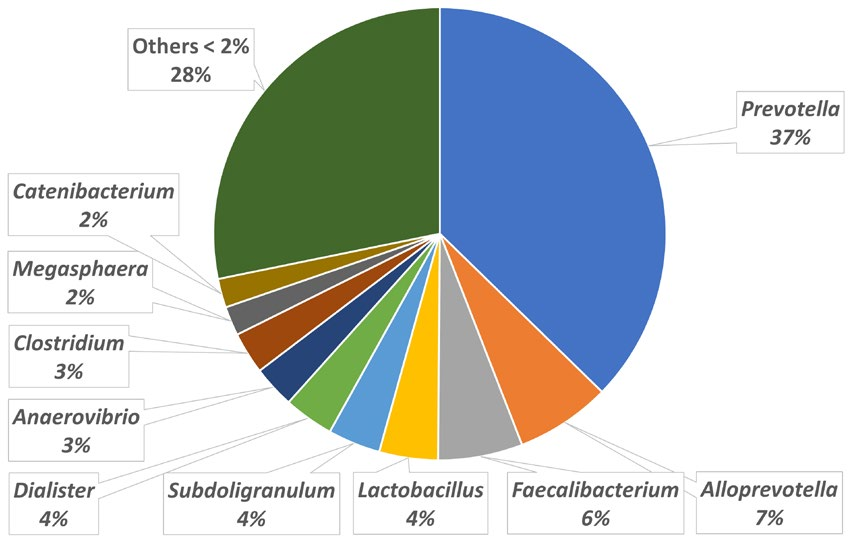
Relative abundances of microbiota at genus level in the caecum.

**Figure 6:**
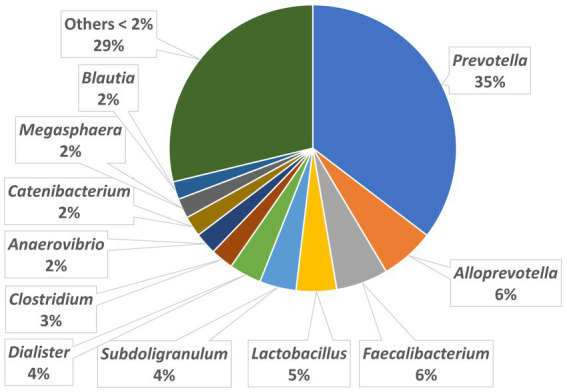
Relative abundances of microbiota at genus level in the colon.

**Figure 7:**
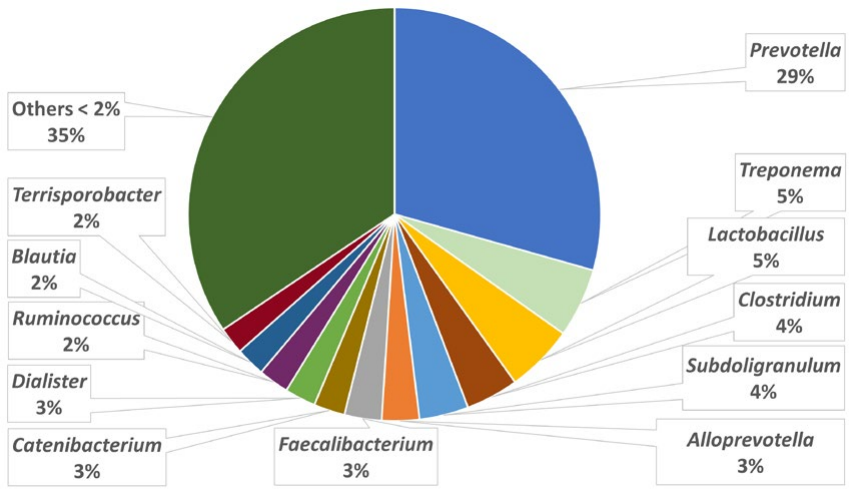
Relative abundances of microbiota at genus level in the feces.

The relative abundances of the highly dominant genera in caecum, colon and feces are presented in Figures 5–7, respectively. The overall microbiota profiles obtained from the samples (caecum, colon, and feces) of all pigs in the trial (stress and control groups) were dominated by the phyla *Firmicutes* and *Bacteroidetes* (cumulatively 90% of the relative abundances). Additionally, the phyla *Spirochaetes, Proteobacteria* and *Actinobacteria* were identified at substantially lower relative abundances (2.6, 2.2, and 2.3%, respectively). At the family level, the overall microbiota was dominated by *Prevotellaceae* (39%), *Ruminococcaceae* (12%), *Lachnospiraceae* (10%), *Veillonellaceae* (9%), and *Erysipelotrichaceae* (6%).

At the genus level, *Prevotella* was dominant in the caecum, colon, and feces, with relative abundances of 37, 35, and 29%, respectively. In the caecum and colon, we observed *Alloprevotella* (relative abundances of 7 and 6%, respectively), *Faecalibacterium* (6 and 6%) and *Lactobacillus* (4 and 5%), whereas in the feces, *Treponema*, *Lactobacillus* and *Clostridium* were the most dominant genera after *Prevotella*, with relative abundances of 5, 5, and 4%, respectively.

### 3.5. Microbiota diversity

We compared the alpha diversity indices of the microbiota between the stress and control groups at each of the three sampling sites (caecum, colon, and feces) (Table 2). The stress group showed a significantly higher richness of genera for all sample sites, in comparison to the control group (*p* < 0.05). Richness was also compared between sampling sites revealing that the caecum had the lowest number of observed genera (G_obs) in both stress and control groups, whereas the colon had the highest number of observed genera in the stress group (average G_obs = 80.53) and the feces had the highest number in the control group (average G_obs = 75.32). Alpha diversity of the microbiota at each site was also assessed by the adjusted Shannon index. Although the adjusted Shannon index was higher due to stress at all sites, non-significant differences were found between stress and control groups, suggesting that the taxonomic profiles at the genus level are similarly evenly distributed between groups. Between sampling sites, the adjusted Shannon index appeared to be significantly higher in feces than in caecum and colon samples for both experimental groups.

**Table 2:** Effect of different sampling sites and the stress treatment on porcine fecal and intestinal microbial diversity indexes within samples (number of observed genera, and adjusted Shannon index) – and across samples (Bray–Curtis dissimilarity).

Diversity index	Group				Sampling site					
		Caecum			Colon			Feces		
		Mean	SE	*p*-value	Mean	SE	*p*-value	Mean	SE	*p*-value
Observed genera^1^ (richness)	Stress	75.89^a^	1.84		80.53^b^	1.51		79.63^a,b^	1.19	
	Control	69.33^a^	1.71		73.37^a,b^	2.09		75.32^b^	1.52	
	Contrast	6.56	2.51	0.01	6.42	2.01	0.003	4.32	1.93	0.03
Adjusted Shannon Index^1^	Stress	0.64^a^	0.01		0.66^a^	0.01		0.70^b^	0.01	
	Control	0.63^a^	<0.01		0.65^a^	0.01		0.69^b^	0.01	
	Contrast	0.02^ns^	0.01	0.12	0.01^ns^	0.01	0.21	0.01^ns^	0.01	0.21
Bray-Curtis dissimilarity^2^	Stress	0.295			0.302			0.398		
	Control	0.265			0.286			0.402		
	Distance between groups	0.295*		0.026	0.306^ns^		0.099	0.430*		0.003

1 The effect of stress treatment on number of observed genera, and adjusted Shannon index was obtained by linear mixed model.

2 The effect of stress treatment on Bray-Curtis dissimilarity was obtained by PERMANOVA.

SE, standard error; *, significant; ns, not significant.

^a,b^Different superscripts within row represent significant differences between sampling sites at *p*<0.05.

Beta-diversity of the microbiota between samples was assessed through the Bray-Curtis index and compared between treatment groups (within sample sites) using PERMANOVA. In caecum samples, a significantly higher dissimilarity between pigs was observed in the stress group, in comparison to control. Regarding the fecal samples, the control group showed significantly higher dissimilarity within the group than their stressed counterparts. In the colon, beta diversity was not significantly different between treatments.

### 3.6. Microbiota biomarkers of social stress

**Figure 8:**
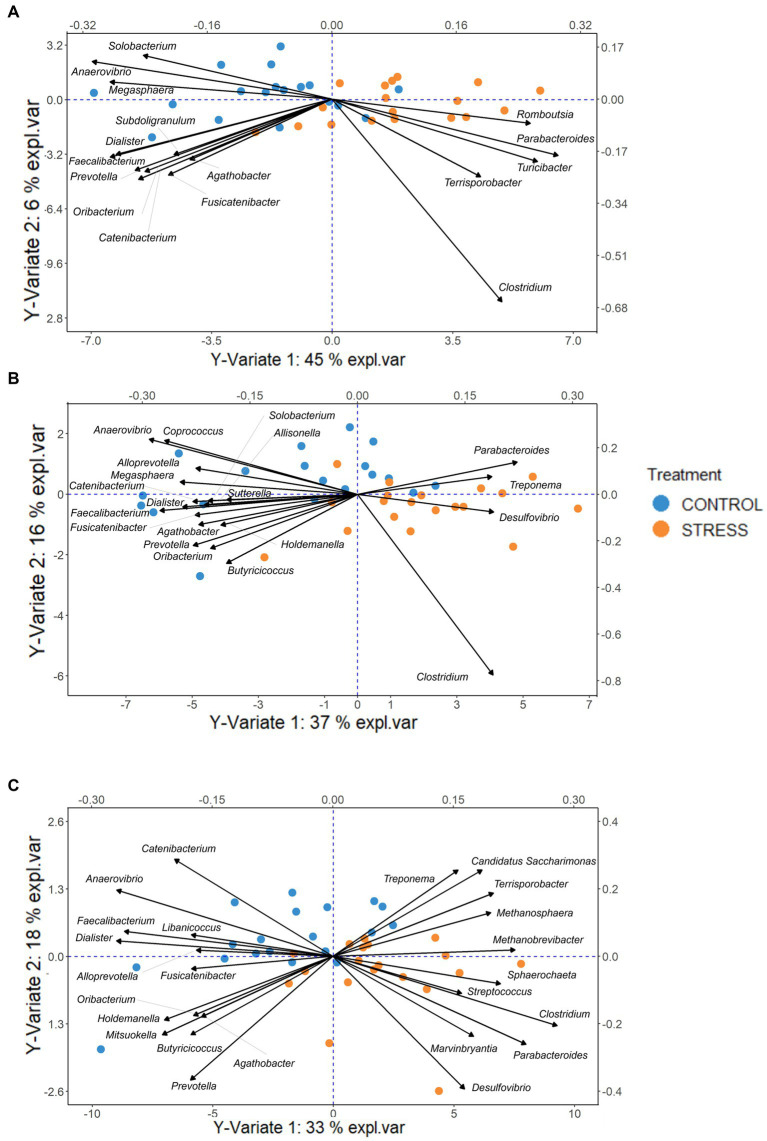
PLSDA biplot, indicating the important genera (VIP > 0.8) discriminative due to stress for each sampling site Caecum **(A)**, Colon **(B)**, Feces **(C)**. The pigs of the stress and control groups were colored orange and blue, respectively. The length of and distance between arrows associated with each important genera indicate the bacteria’s strongness for the discrimination and the correlation between them.

The PLSDA models for discrimination of stressed animals from the control resulted in successful classification rates of 81, 74, and 76% based on microbiota profiles of the caecum, colon, and feces, respectively. The biplots (including samples and microbial genera) for each of the three sampling sites revealed that most of the animals of the stress and control groups are clustered to the right and left sites within Figure 8.

**Figure 9:**
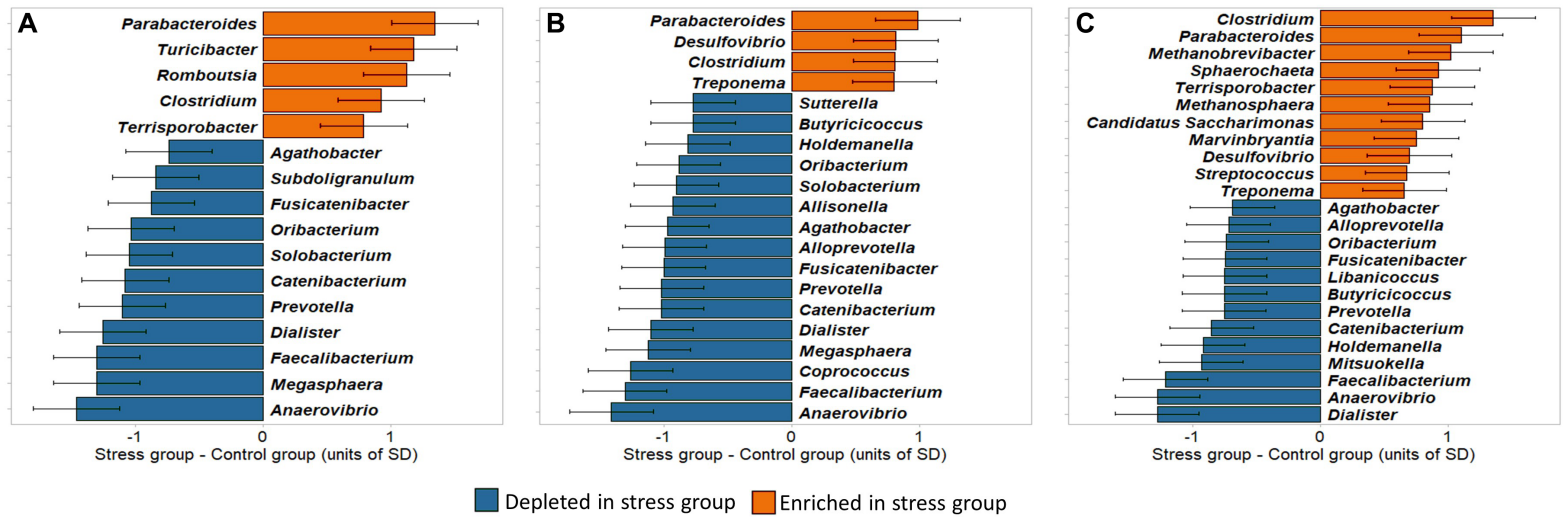
Differences between stress and control groups in *clr*-transformed abundances of microbial genera identified to be important for discrimination by the PLSDA model. Differences are expressed in units of standard deviations. **(A)** Caecum, **(B)** Colon; **(C)** Feces.

By combining the PLSDA results with Welsh’s *t*-test, we explored whether the identified genera were significantly enriched or depleted in the stress group in comparison with the control group. To facilitate the interpretation, the differences between stress and control groups in *clr*-transformed abundances were expressed in units of standard deviations of the traits, shown in Figure 9.

Our results showed that, in all sampling sites, the largest abundance differences between stress and control, i.e., exceeding 1 unit of standard deviation, were found in *Parabacteroides* (more abundant in the stress group), and *Anaerovibrio*, *Faecalibacterium*, and *Dialister* (more abundant in the control group).

In the caecum, 16 bacterial taxa were found to be significantly different between groups (*p* < 0.05), comprising 14 *Firmicutes* and 2 *Bacteroidetes* phyla. Five of these (the *Bacteroidetes Parabacteroides*, and the *Firmicutes Turicibacter, Romboutsia, Clostridium, Terrisporobacter*) were enriched and 11 (the *Bacteroidetes Prevotella*, and the *Firmicutes Anaerovibrio, Faecalibacterium, Dialister, Catenibacterium, Oribacterium, Fusicatenibacter, Agathobacter, Megasphaera, Solobacterium, Subdoligranulum*) were depleted in the stress in comparison to the control group.

Of the 16 bacterial genera in the caecum identified as stress biomarkers, 12 were also found to be significantly different between the colon samples of the two treatment groups. In the colon samples 8 further significant genera were identified, 6 depleted (*Alloprevotella*, *Butyricicoccus*, *Coprococcus*, *Allisonella*, *Holdemanella*, and *Sutterella*) and 2 enriched (*Treponema* and *Desulfovibrio*), in stress group in comparison to control.

In the feces, 24 genera were identified as potential biomarkers for social stress, of which 16 overlapped with those identified in the colon or caecum. Within the 8 genera exclusively identified in feces, *Libanicoccus* and *Mitsuokella* were depleted, whereas *Streptococcus*, *Marvinbryantia*, *Candidatus Saccharimonas*, *Methanosphaera*, *Sphaerochaeta*, and *Methanobrevibacter* were enriched in the stress group. Most of the stress-enriched genera identified in the feces belonged to the same phyla as those enriched in the caecum and colon (*Bacteroidetes* and *Firmicutes*), and 4 other genera belonged to phyla *Actinobacteria*, *Euryarchaeota*, and *Patescibacteria.* The Welsh’s *t*-test then showed that most of them were significantly different between treatment groups [exceptions were *Butyricicoccus* (*p* = 0.0618), *Marvinbryantia* (*p* = 0.0566), *Desulfovibrio* (*p* = 0.0973), *Agathobacter* (*p* = 0.0610), *Streptococcus* (*p* = 0.0757), and *Treponema* (*p* = 0.0736)] which approached the significance level (Supplementary Tables S1–S3).

### 3.7. Porcine pathogen resistance

Based on the relative abundances of opportunistic pathogenic bacteria such as *Clostridium*, *Treponema*, *Streptococcus*, and *Campylobacter,* we observed that some animals are more susceptible to the growth of these pathogens, whereas others were very resistant, preventing large multiplication of these pathogens even under stress (Figure 2). We classified those samples presenting a relative abundance greater than 2.5 times the group median (computed within stress or control groups) as susceptible. Susceptible animals had higher abundances of the 4 pathogenic genera across all sampling sites than resistant animals with the posterior probabilities of the differences being positive (Pr0) equal 100% (Supplementary Table S4).

Including additionally the resistance/susceptibility effect into the stress/control model showed even when a few animals were susceptible to pathogen being a highly probable effect (Pr0 ≥ 96%). After adjustment for differences in resistance/susceptibility of pigs to pathogens within treatment, significant differences between stress and control groups were obtained in the *clr*-transformed abundances of *Clostridium* and *Treponema* in all locations, and for *Streptococcus* in feces (Pr0 ≥ 98%). For *Campylobacter*, pigs in the stress group tended to have higher average abundances than the control group, however the probabilities of being enriched were at 69, 95, and 69% for caecal, colonic and fecal data, respectively (Supplementary Table S4).

## 4. Discussion

Our results suggest that social stress has a substantial negative impact on several aspects of pig welfare (increased lesions) and productivity, leading to reduced feed intake and growth rate as well as alterations in the caecal, colonic, and fecal microbiota profiles.

Our porcine social stress model successfully increased the release of cortisol in saliva cumulatively over time suggesting that the severity of the stress increased throughout the experiment. This increase suggested that alterations in the HPA activity, the main stress response axis of the body (Smith and Vale, 2006), were persistent throughout the experiment. This finding was in agreement with previous studies that showed increased cortisol concentration in pigs exposed to different stressors, such as shipping (McGlone et al., 1993), altered ambient temperature (Hicks et al., 1998), and social stress (Rutherford et al., 2006; Escribano et al., 2015; Casal et al., 2017). One outcome of the social stress model was the substantial increase in skin lesions, most likely due to the regrouping of animals, which caused vigorous physical aggression between unfamiliar individuals, potentially because a new hierarchy had to be repeatedly established (Desire et al., 2015a; Foister et al., 2018). Skin lesions are related to animal welfare and are also influenced by animal genetics (Desire et al., 2015b; Agha et al., 2022).

The stress condition led to reductions of 12.4% in the voluntary feed intake, 16.1% in the growth rate, and 5.9% in feed use efficiency in comparison to the controls, which may lead to a delay in reaching the slaughter weight, negatively impacting productivity. Hyun et al. (1998) reported decreased growth rates of 7.1 and 15.7% in pigs subject to regrouping and crowding, respectively, but did not find significant differences in voluntary feed intake between treatment groups.

The impact of stress on the intestinal and fecal microbiome adjusted Shannon index was not significant. However, animals in the stress group had microbiota profiles at the genera level with significantly higher richness than the control animals. The beta-diversity differences observed between the stress and control groups were not consistent throughout the sample collection sites. A negative control sample (containing no DNA) and a positive mock control sample with a known bacterial composition (in duplicate) alongside the experimental samples were not available and therefore could not be used to get further insight into the accuracy of the sequencing results. These findings are consistent with the review of Kuo and Chung (2019), in which the authors concluded that the impact of neuro-psychiatric conditions and depression on the stability of human microbiome diversity is inconsistent throughout the literature. Likewise, in a study on physically healthy women, the fecal microbiome diversity of participants with anxiety and depression was not significantly different from that of the psychologically healthy woman (Kleiman et al., 2017). Michels et al. (2019) claimed that even though higher diversity is often recognized as a good health indicator, its association with the brain’s function and health remains unclear.

When interpreting the results, it has to be considered that stressed pigs may have encountered a higher bacterial load due to weekly mixing, potentially contributing to larger microbiota richness in the intestine compared to the control group. However, the control pigs were kept in the same room so that due to ventilation the different exposure may not be substantially different. In addition, pigs from each litter were distributed across experimental groups, ensuring a balanced early-life microbial exposure. Furthermore, the experiment was based on grown up pigs in the final 4 weeks of their life so that different microbial exposure due to mixing may be of negligible impact on the intestinal microbiota. Generally, it is reported that a stress impaired immune system is closely linked to intestinal microbiota profiles (Gimsa et al., 2018). Hence, the observed larger microbiota richness in the stressed group is most likely attributed to this stress-induced immune modulation.

We identified 32 genera whose abundances differed significantly between the stress and control groups across all sampling sites, and 10 of them were identified in all locations (*Anaerovibrio, Faecalibacterium, Dialister, Prevotella, Catenibacterium, Oribacterium, Fusicatenibacter, Agathobacter, Clostridium*, and *Parabacteroides*). Five out of seven stress-enriched biomarkers that were identified in the caecum and colon were also identified in the feces, suggesting that the feces represent the most suitable sample type for microbiota-based studies on stress in pigs, which is advantageous given the ease of access to this sample type.

We have identified the genus *Clostridium* to be significantly enriched in stressed animals consistently over all sample sites. Some *Clostridium* species are part of the commensal porcine core microbiota, and they are involved in saccharolytic and proteolytic metabolism (Holman et al., 2017). However, *Clostridium perfringens* types A and C and *Clostridium difficile* are opportunistic pathogens that can cause significant enteric porcine infections (Songer and Uzal, 2005) and have been found to severely infect hospitalized humans (Kelly and LaMont, 1998).

Our results agree with previous observations of increased relative abundance of *Terrisporobacter*, *Marvinbryantia* and *Romboutsia* (all belonging to the *Clostridiales* order, such as *Clostridium*) in stressed animals. Whereas *Terrisporobacter* and *Marvinbryantia* belong to the *Peptostreptococcaceae* family, which has previously been observed to be enriched in patients with *Clostridium difficile* infection (Stewart et al., 2019), *Romboutsia* was increased in mice with chronic unpredicted mild stress (Sun et al., 2019). The authors found that *Romboutsia’*s abundance was positively correlated with mice anxiety- and depression-like behaviors.

*Streptococcus, Parabacteroides* and *Desulfovibrio* were also enriched in pigs in the stress group. In humans, *Streptococcus* and *Desulfovibrio* have been reported as increased in major depressive disorder patients (Simpson et al., 2021), and may be involved in the modulation of inflammatory response in depression patients (Evrensel and Ceylan, 2015; Barandouzi et al., 2020). *Desulfovibrio* and its product hydrogen sulfide were found to contribute to inflammation modulating and to the development of different inflammatory bowel diseases (Loubinoux et al., 2002; Bisson-Boutelliez et al., 2010; Kushkevych et al., 2018, 2021). Kverka et al. (2011) found that orally fed membranous fraction of *Parabacteroides distasonis* lysate to dextran sulfate sodium-induced colitis mice reduced the production of both proinflammatory and anti-inflammatory cytokines in the colon of treated mice. Considering that the relative abundances of these two genera were positively correlated through the three sampling sites, the inflammatory modulation effect can be the common function that led to the strong associations between them.

*Treponema* (a known opportunistic pathogen) was enriched in the stress group (colon and feces samples) and is therefore proposed as a potential biomarker for stress in pigs. *Treponema* is associated with porcine colonic spirochetosis, a diarrheal disease resulting in hindered performance. *Treponema hyodysenteriae*, recently reclassified as *Brachyspira hyodysenteriae*, can cause swine dysentery (Burrough, 2017). Another species, *Treponema pallidum*, is linked to diarrheal illness in pigs, as well as in humans (Tsinganou and Gebbers, 2010).

The enrichment of *Turicibacter* in the stress group is interesting because this may be due to the pig’s ability to adapt to stress. The relative abundances of *Turicibacter* in the pigs’ intestinal samples were highly consistent with the correlation of 0.88 between caecal and colonic datasets whereas they were not significantly associated with salivary cortisol (*p* > 0.05). These findings suggested an involvement of another mechanism led to the enrichment. In a recent study in mice, Fung et al. (2019) discovered that *Turicibacter sanguinis* can co-evolve with the host to induce serotonin production. Serotonin is a neurotransmitter involved in many aspects of neural activities, such as stabilizing mood, social behaviors and increasing happiness. The authors found that *T. sanguinis* had a special receptor named CVW-0748 that was structurally and functionally similar to the serotonin transporter of the host. As a result, *T. sanguinis* could uptake the host’s serotonin and then increase in abundance in the intestine. Fung et al. (2019) also demonstrated the bidirectional microbiome-gut-brain axis and recommended further investigations into the spore-forming bacteria and gut-derived serotonin (Hoffman and Margolis, 2020), especially in stress conditions as suggested by the results in growing pigs in this study. Moreover, *T. sanguinis* is involved in the regulation of the host’s steroid and lipid metabolism and is reported to be associated with Parkinson’s disease (Jin et al., 2019) and depression (Jackson et al., 2018).

Even though *Sphaerochaeta* belongs to the same family as *Treponema* (*Spirochaetacea*), it does not have the two key features of pathogenic spirochaetes, i.e., helical or spiral morphology and motility (Ritalahti et al., 2011; Miyazaki et al., 2014; Dong et al., 2018). Caro-Quintero et al. (2012) found that *Sphaerochaeta* genomes have relatively higher numbers of fermentation and carbohydrate metabolism genes than other spirochetes, promoting a fermentative lifestyle. Their increase is expected to have no negative effects on the host, suggesting that even commensal bacteria in the porcine gastrointestinal tract were influenced by stress.

The bacterium *Candidatus Saccharimonas,* and the archaea *Methanosphaera* and *Methanobrevibacter* were also enriched due to social stress. These bacteria may have a role in degrading fiber and gut microbiota stabilization (Chaucheyras-Durand et al., 2010; Albertsen et al., 2013; Kindaichi et al., 2016; Mi et al., 2019). Interestingly, the two methane-production genera *Methanosphaera* and *Methanobrevibacter* were plotted relatively close to each other, reflecting their positive correlation (Figure 8).

One highlight of our research is that many bacteria reported to be positively related to health were depleted in the stress group, including *Prevotella, Faecalibacterium, Butyricicoccus, Dialister, Megasphaera*, and *Mitsuokella*. *Prevotella* is a dominant population in the porcine intestine and metabolizes hemicelluloses and pectin to acetate, an energy supply for the host and other bacteria. *Faecalibacterium* and *Butyricicoccus* produce butyrate (Duncan et al., 2004) which is a key energy source for colonocytes and has potent anti-inflammatory properties, thus benefiting host health (Hamer et al., 2007; van der Beek et al., 2017).

Recently, Valles-Colomer et al. (2019) found that *Faecalibacterium* were positively associated with a majority of the quality of life scores in the RAND-36 Health Survey (Hays and Morales, 2001), including both mental and physical aspects, such as social functioning, emotional well-being, vitality, and physical functioning. In addition, *Dialister* was significantly positively associated with the physical RAND scores and significantly depleted in depression patients (Valles-Colomer et al., 2019). In our study, *Faecalibacterium* and *Dialister* were in all sampling sites found to be depleted in the stress group and strongly associated with each other. *Coprococcus, Agathobacter*, *Fusicatenibacter* and *Oribacterium* are from the *Lachnospiraceae* family, and the latter three genera are closely associated with each other. *Lachnospiraceae* was previously reported to be depleted in patients with major depressive disorder (Jiang et al., 2015). Similarly, *Coprococcus* (Liu et al., 2016; Huang et al., 2018; Valles-Colomer et al., 2019), *Fusicatenibacter* (Chen et al., 2021) and *Agathobacter* (Hua et al., 2020; Chen et al., 2021) showed negative associations with depression symptoms. These results suggest that the imposed social stress in pigs affects the same bacteria associated with depression and quality of life in humans. Generally, these findings provide evidence of many similarities in the microbiota-gut-brain axis in humans and pigs.

*Dialiste*r and the *Anaerovibrio, Megasphaera*, *Mitsuokella* and *Allisonella* are all belonging to the family of *Veillonellaceae* and were all depleted in the stress group. *Megasphaera elsdenii* is known to reduce acidosis in ruminants by metabolizing lactate to SCFA. In a caecal *in-vitro* porcine model *Megasphaera elsdenii* was shown to substantially increase the production of butyrate supporting its beneficial health effects and therefore was suggested as a probiotic agent (Tsukahara et al., 2006). Two novel *Megasphaera* species isolated from the human gut had diverse and unique sets of Carbohydrate-Active enzymes (CAZymes), that produced SCFA, vitamins and essential amino acids which further promote the potential health effects on the host (Shetty et al., 2013). *Mitsuokella jalaludinii* isolated from the porcine gut was found to inhibit *Salmonella* growth and invasion due to the production of SCFA and reduction in pH (Levine et al., 2012).

SCFA have anti-inflammatory effects (Parada Venegas et al., 2019), therefore their decrease may directly contribute to the potential microbiota-mediated inflammation in the anxiety/depression groups in humans (Simpson et al., 2021). In this study, SCFA-producing genera were depleted in the stress group, such as *Catenibacterium*, *Fusicatenibacter* (Takada et al., 2019), *Agathobacter* (Rosero et al., 2016), *Alloprevotella* (Downes et al., 2013), *Butyricicoccus* (Eeckhaut et al., 2008), *Subdoligranulum* (Holmstrøm et al., 2004). Our results indicate a correlation between the abundance of these genera (clustered together in the PLSDA biplots, Figure 8), which might be explained by their common role in SCFA production.

The impact of commensal genera *Sutterella*, *Libanicoccus*, and *Holdemanella* on porcine health and stress are unclear. *Sutterella* is reported to be commensal with high prevalence in the human gut. It has minimal impact on epithelial homeostasis and is possibly involved in regulating the gut immune system (Hiippala et al., 2016). *Libanicoccus* and *Holdemanella* are identified in the human gut and feces, but research on their impact on human and porcine physical and mental health is still lacking (Bilen et al., 2018). The depletion of these three genera in the stress group was probably due to changes in host immune response, feed intake and efficiency.

Our results support the hypothesis that stress altered the intestinal and fecal microbiota profiles. However, the stress group also showed decreased voluntary feed intake. This suggests that stress may have directly influenced the microbiota profiles, or that this influence occurred in an indirect manner, due to changes in feed intake that led to alterations in the microbiota, or even both simultaneously. Diet is one of the most important factors influencing the gastrointestinal microbiota, since changes in nutrient availability will favor or impair/inhibit the growth of microbial communities. However, the composition of the diet did not change, so only less nutrients of the same composition were available in the stressed animals. Therefore, we hypothesis that the change in microbiota profiles as a results of stress are mainly due to the microbiota-gut-brain axis, supported by the substantial higher salivary cortisol concentration in the stress in comparison to the control group (Misiak et al., 2020).

Based on the low abundance of the opportunistic pathogenic bacteria *Clostridium*, *Treponema* and *Streptococcus* in the gut of most of the experimental animals even under stress and relatively few animals with extremely large abundances, we hypothesize that most of the animals in this experiment were resistant to the growth of these pathogens. Resistance against these pathogens, which might be explained by host-genetic factors, is an important attribute in livestock production (Bishop and Woolliams, 2014; Bai et al., 2020) to help animals maintain their performance with minimal treatment. This resistance might be also beneficial for pigs while adapting to challenging social stressors. Breeding for improved adaptation to stressors has been previously discussed, such as selection for higher heat tolerance to adapt to heat stress in tropical climate (Gourdine et al., 2019) or higher resistance to reduce disease risk and prevalence (Tsairidou et al., 2019). The identification of biomarkers indicating the extent to which an animal is protected against pathogen growth in the gut, in particular under stress conditions, therefore is of great interest for supporting breeding for the resistance of pigs.

*Campylobacter* is one of the leading food-borne zoonoses worldwide (Havelaar et al., 2015), and *Campylobacter coli* is the main species in pigs that cause gastrointestinal infection in humans (Fosse et al., 2009). *Campylobacter* infection in humans is the most frequently reported gastrointestinal disease in Europe with an estimated annual cost of 2.4 billion Euros (Elliott et al., 2012; EFSA and ECDC, 2018). Whereas this bacterium causes gastroenteritis in humans, pigs do not show any symptoms. In this study, there was a tendency of the enrichment of *Campylobacter* in the stress group; however, the probabilities of being enriched were relatively low at 59, 94 and 65% in caecum, colon and feces, respectively.

## 5. Conclusion

Our porcine stress model with weekly regrouping of animals and reduced space allowance during the last 4 weeks of the finishing period was shown to be effective, based on the sharp increase in stress-induced salivary cortisol concentration, which gradually increased throughout the experimental period. These changes in salivary cortisol levels reflect HPA activity as the major stress response axis. Although there are differences in stress-induced bacterial biomarkers between sampling sites, most of the important stress biomarkers were identified in feces. Opportunistic pathogenic genera such as *Clostridium* and *Treponema* were significantly enriched due to social stress, demonstrating that stress impairs the ability of animals to defend against pathogenic bacteria, and confirming the influence of the HPA axis on the composition of the gut microbiota. In particular, the distribution of abundances of the opportunistic pathogenic bacteria *Treponema* and *Campylobacter* indicates the differential ability of animals to cope with stress and, assuming a genetic component, should be investigated as a selection criterion for breeding for resistance to stress-induced pathogenic bacteria growth in the gut. Bacteria that are depleted in the stress group are very informative, as they have previously been shown to be associated with health benefits in various animal species due to their metabolic products, especially SCFA, which might be involved in the inhibition of the growth of pathogenic bacteria. Of particular interest are the bacteria *Dialister* and *Faecalibacterium*, which were reduced by social stress in our porcine study and depleted in humans with depression and low quality of life. Therefore, bacteria depleted by social stress in our study provide biomarkers to identify stress and may potentially be used as probiotic agents to reduce the effects of stress on host health. In addition, *Turicibacter* enriched due to stress is a highly interesting candidate biomarker to explore antidepressant probiotics. In general, our results confirm the microbiota-gut-brain axis communication as demonstrated by increased cortisol levels regulated by the HPA axis and the alteration of microbiota composition, particularly bacteria known to be associated with poor mental health.

## Data availability statement

The data presented in the study are deposited in the European Nucleotide Archive repository, at: https://www.ebi.ac.uk/ena, accession number PRJEB60501.

## Ethics statement

The animal study was approved by SRUC’s Animal Welfare and Ethical Approval Body. The study was conducted in accordance with the local legislation and institutional requirements.

## Author contributions

RR, KR, and MA: conceptualisation. TN: statistical analysis and writing original draft. TN, MM-Á, JL, and RR: formal analysis, methodology, and software. TN, MM-Á, JL, MA, KR, GS, RD, and RR: investigation and validation. TN, MM-Á, JL, MA, KR, GS, RD, EB, and RR: review and editing. All authors contributed to the article and approved the submitted version.

## References

[ref9001] Agha S. Foister S. Roehe R. Turner S. P. Doeschl-Wilson A. (2022). Genetic Analysis of Novel Behaviour Traits in Pigs Derived from Social Network Analysis. Genes. 13:561. doi: 10.3390/genes13040561 35456367 PMC9027576

[ref1] Albertsen M. Hugenholtz P. Skarshewski A. Nielsen K. L. Tyson G. W. Nielsen P. H. (2013). Genome sequences of rare, uncultured bacteria obtained by differential coverage binning of multiple metagenomes. Nat. Biotechnol. 31, 533–538. doi: 10.1038/nbt.2579 23707974

[ref2] Andersen I. L. Nævdal E. Bakken M. Bøe K. E. (2004). Aggression and group size in domesticated pigs, *Sus scrofa*: When the winner takes it all and the loser is standing small. Anim. Behav. 68, 965–975. doi: 10.1016/j.anbehav.2003.12.016

[ref3] Bai X. Putz A. M. Wang Z. Fortin F. Harding J. C. S. Dyck M. K.. (2020). Exploring phenotypes for disease resilience in pigs using complete blood count data from a natural disease challenge model. Front. Genet. 11:216. doi: 10.3389/fgene.2020.00216 32231686 PMC7083204

[ref4] Bailey M. T. Dowd S. E. Galley J. D. Hufnagle A. R. Allen R. G. Lyte M. (2011). Exposure to a social stressor alters the structure of the intestinal microbiota: Implications for stressor-induced immunomodulation. Brain Behav. Immun. 25, 397–407. doi: 10.1016/j.bbi.2010.10.023 21040780 PMC3039072

[ref5] Barandouzi Z. A. Starkweather A. R. Henderson W. A. Gyamfi A. Cong X. S. (2020). Altered composition of gut microbiota in depression: a systematic review. Front. Psych. 11:541. doi: 10.3389/fpsyt.2020.00541 PMC7299157 32587537

[ref6] Bates D. Mächler M. Bolker B. Walker S. (2015). fitting linear mixed-effects models using lme4. J. Stat. Softw. 67, 1–48. doi: 10.18637/jss.v067.i01

[ref7] Bilen M. Cadoret F. Richez M. Tomei E. Daoud Z. Raoult D.. (2018). *Libanicoccus massiliensis* gen. nov., sp. nov., a new bacterium isolated from human stool. New Microbes New Infect. 21, 63–71. doi: 10.1016/j.nmni.2017.11.001 29204287 PMC5711663

[ref8] Bishop S. C. Woolliams J. A. (2014). Genomics and disease resistance studies in livestock. Livest. Sci. 166, 190–198. doi: 10.1016/j.livsci.2014.04.034 26339300 PMC4547482

[ref9] Bisson-Boutelliez C. Massin F. Dumas D. Miller N. Lozniewski A. (2010). Desulfovibrio spp. survive within KB cells and modulate inflammatory responses. Mol Oral Microbiol 25, 226–235. doi: 10.1111/j.2041-1014.2009.00550.x 20536750

[ref10] Bohórquez D. V. Liddle R. A. (2015). The gut connectome: Making sense of what you eat. J. Clin. Invest. 125, 888–890. doi: 10.1172/JCI81121 25729849 PMC4382222

[ref11] Bohórquez D. V. Samsa L. A. Roholt A. Medicetty S. Chandra R. Liddle R. A. (2014). An enteroendocrine cell – enteric glia connection revealed by 3D electron microscopy. PLoS One 9:e89881. doi: 10.1371/journal.pone.0089881 24587096 PMC3935946

[ref12] Bürkner P. C. (2021). Bayesian item response modeling in R with brms and Stan. J. Stat. Softw. 100, 1–54. doi: 10.18637/JSS.V100.I05

[ref13] Burrough E. R. (2017). Swine dysentery. Vet. Pathol. 54, 22–31. doi: 10.1177/0300985816653795 27288432

[ref14] Callahan B. J. McMurdie P. J. Rosen M. J. Han A. W. Johnson A. J. A. Holmes S. P. (2016). DADA2: High-resolution sample inference from Illumina amplicon data. Nat. Methods 13, 581–583. doi: 10.1038/nmeth.3869 27214047 PMC4927377

[ref15] Cannon W. B. (1929). Bodily changes in pain, hunger, fear and rage. New York: Appleton.

[ref16] Caro-Quintero A. Ritalahti K. M. Cusick K. D. Löffler F. E. Konstantinidis K. T. (2012). The chimeric genome of sphaerochaeta: nonspiral spirochetes that break with the prevalent dogma in spirochete biology. MBio 3, e00025–e00012. doi: 10.1128/mBio.00025-12 22589287 PMC3372971

[ref17] Casal N. Manteca X. Peña L. R. Bassols A. Fàbrega E. (2017). Analysis of cortisol in hair samples as an indicator of stress in pigs. J. Vet. Behav. 19, 1–6. doi: 10.1016/j.jveb.2017.01.002

[ref18] Chaucheyras-Durand F. Masséglia S. Fonty G. Forano E. (2010). Influence of the composition of the cellulolytic flora on the development of hydrogenotrophic microorganisms, hydrogen utilization, and methane production in the rumens of gnotobiotically reared lambs. Appl. Environ. Microbiol. 76, 7931–7937. doi: 10.1128/AEM.01784-10 20971877 PMC3008260

[ref19] Chen Y. Xue F. Yu S. Li X. Liu L. Jia Y.. (2021). Gut microbiota dysbiosis in depressed women: The association of symptom severity and microbiota function. J. Affect. Disord. 282, 391–400. doi: 10.1016/j.jad.2020.12.143 33421868

[ref20] Cornale P. Macchi E. Miretti S. Renna M. Lussiana C. Perona G.. (2015). Effects of stocking density and environmental enrichment on behavior and fecal corticosteroid levels of pigs under commercial farm conditions. J. Vet. Behav. Clin. Appl. Res. 10, 569–576. doi: 10.1016/j.jveb.2015.05.002

[ref21] Coutellier L. Arnould C. Boissy A. Orgeur P. Prunier A. Veissier I.. (2007). Pig’s responses to repeated social regrouping and relocation during the growing-finishing period. Appl. Anim. Behav. Sci. 105, 102–114. doi: 10.1016/j.applanim.2006.05.007

[ref22] Cryan J. F. O’Mahony S. M. (2011). The microbiome-gut-brain axis: From bowel to behavior. Neurogastroenterol. Motil. 23, 187–192. doi: 10.1111/j.1365-2982.2010.01664.x 21303428

[ref23] D’Allaire S. Drolet R. Brodeur D. (1996). Sow mortality associated with high ambient temperatures. Can. Vet. J. 37, 237–239. 8801022 PMC1576357

[ref24] D’Eath R. B. (2002). Individual aggressiveness measured in a resident-intruder test predicts the persistence of aggressive behaviour and weight gain of young pigs after mixing. Appl. Anim. Behav. Sci. 77, 267–283. doi: 10.1016/S0168-1591(02)00077-1

[ref25] Desire S. Turner S. P. D’Eath R. B. Doeschl-Wilson A. B. Lewis C. R. G. Roehe R. (2015a). Analysis of the phenotypic link between behavioural traits at mixing and increased long-term social stability in group-housed pigs. Appl. Anim. Behav. Sci. 166, 52–62. doi: 10.1016/j.applanim.2015.02.015

[ref9002] Desire S. Turner S. P. D’Eath R. B. Doeschl-Wilson A. B. Lewis C. R. G. Roehe R. (2015b). Genetic associations of short- and long-term aggressiveness identified by skin lesion with growth, feed efficiency, and carcass characteristics in growing pigs. J. Anim. Sci. 93, 3303–3312. doi: 10.2527/jas.2014-8823 26439999

[ref26] Dong X. Greening C. Brüls T. Conrad R. Guo K. Blaskowski S.. (2018). Fermentative Spirochaetes mediate necromass recycling in anoxic hydrocarbon-contaminated habitats. ISME J. 12, 2039–2050. doi: 10.1038/s41396-018-0148-3 29849169 PMC6052044

[ref27] Downes J. Dewhirst F. E. Tanner A. C. R. Wade W. G. (2013). Description of Alloprevotella rava gen. nov., sp. nov., isolated from the human oral cavity, and reclassification of *Prevotella tannerae* Moore et al. 1994 as Alloprevotella tannerae gen. nov., comb. nov. Int. J. Syst. Evol. Microbiol. 63, 1214–1218. doi: 10.1099/ijs.0.041376-0 22753527 PMC3709537

[ref28] Duncan S. H. Louis P. Flint H. J. (2004). Lactate-utilizing bacteria, isolated from human feces, that produce. Appl. Environ. Microbiol. 70, 5810–5817. doi: 10.1128/AEM.70.10.5810 15466518 PMC522113

[ref29] Eeckhaut V. Van Immerseel F. Teirlynck E. Pasmans F. Fievez V. Snauwaert C.. (2008). *Butyricicoccus pullicaecorum* gen. nov., sp. nov., an anaerobic, butyrate-producing bacterium isolated from the caecal content of a broiler chicken. Int. J. Syst. Evol. Microbiol. 58, 2799–2802. doi: 10.1099/ijs.0.65730-0 19060061

[ref30] EFSA and ECDC (2018). The European Union summary report on trends and sources of zoonoses, zoonotic agents and food-borne outbreaks in 2017. EFSA J. 16:e05500. doi: 10.2903/j.efsa.2018.5500 32625785 PMC7009540

[ref31] Elliott J. Lee D. Erbilgic A. Jarvis A. (2012). Analysis of the costs and benefits of setting certain control measures for reduction of Campylobacter in broiler meat at different stages of the food chain. ICF GHK in Association with ADAS, London, 105.

[ref32] Escribano D. Gutiérrez A. M. Tecles F. Cerón J. J. (2015). Changes in saliva biomarkers of stress and immunity in domestic pigs exposed to a psychosocial stressor. Res. Vet. Sci. 102, 38–44. doi: 10.1016/j.rvsc.2015.07.013 26412517

[ref33] Evrensel A. Ceylan M. E. (2015). The gut-brain axis: The missing link in depression. Clin. Psychopharmacol. Neurosci. 13, 239–244. doi: 10.9758/cpn.2015.13.3.239 26598580 PMC4662178

[ref34] Fernandes A. D. Macklaim J. M. Linn T. G. Reid G. Gloor G. B. (2013). ANOVA-like differential expression (ALDEx) analysis for mixed population RNA-Seq. PLoS One 8:e67019. doi: 10.1371/journal.pone.0067019 23843979 PMC3699591

[ref35] Fernandes A. D. Reid J. N. Macklaim J. M. McMurrough T. A. Edgell D. R. Gloor G. B. (2014). Unifying the analysis of high-throughput sequencing datasets: characterizing RNA-seq, 16S rRNA gene sequencing and selective growth experiments by compositional data analysis. Microbiome 2:15. doi: 10.1186/2049-2618-2-15 24910773 PMC4030730

[ref36] Foister S. Doeschl-Wilson A. Roehe R. Arnott G. Boyle L. Turner S. (2018). Social network properties predict chronic aggression in commercial pig systems. PLoS One 13:e0205122. doi: 10.1371/journal.pone.0205122 30286157 PMC6171926

[ref37] Fosse J. Seegers H. Magras C. (2009). Prevalence and risk factors for bacterial food-borne zoonotic hazards in slaughter pigs: a review. Zoonoses Public Health 56, 429–454. doi: 10.1111/j.1863-2378.2008.01185.x 19175574 PMC7165994

[ref38] Fung T. C. Vuong H. E. Luna C. D. G. Pronovost G. N. Aleksandrova A. A. Riley N. G.. (2019). Intestinal serotonin and fluoxetine exposure modulate bacterial colonization in the gut. Nat. Microbiol. 4, 2064–2073. doi: 10.1038/s41564-019-0540-4 31477894 PMC6879823

[ref39] Giersing M. Andersson A. (1998). How does former acquaintance affect aggressive behaviour in repeatedly mixed male and female pigs? Appl. Anim. Behav. Sci. 59, 297–306. doi: 10.1016/S0168-1591(98)00141-5

[ref40] Gimsa U. Tuchscherer M. Kanitz E. (2018). Psychosocial stress and immunity—what can we learn from pig studies? Front. Behav. Neurosci. 12, 1–9. doi: 10.3389/fnbeh.2018.00064 29666573 PMC5891618

[ref41] Goumon S. Faucitano L. (2017). Influence of loading handling and facilities on the subsequent response to pre-slaughter stress in pigs. Livest. Sci. 200, 6–13. doi: 10.1016/j.livsci.2017.03.021

[ref42] Gourdine J. L. Riquet J. Rosé R. Poullet N. Giorgi M. Billon Y.. (2019). Genotype by environment interactions for performance and thermoregulation responses in growing pigs. J. Anim. Sci. 97, 3699–3713. doi: 10.1093/jas/skz245 31351442 PMC6735898

[ref43] Graham H. Åman P. (1987). The pig as a model in dietary fibre digestion studies. Scand. J. Gastroenterol. 22, 55–61. doi: 10.3109/00365528709095851 2820046

[ref44] Greenacre M. (2018). Compositional Data Analysis in Practice. New York: Chapman and Hall/CRC Available at: https://www.taylorfrancis.com/books/9780429849022.

[ref45] Grenham S. Clarke G. Cryan J. F. Dinan T. G. (2011). Brain-gut-microbe communication in health and disease. Front. Physiol. 2 DEC, 1–15. doi: 10.3389/fphys.2011.00094 22162969 PMC3232439

[ref46] Guevarra R. B. Hong S. H. Cho J. H. Kim B. R. Shin J. Lee J. H.. (2018). The dynamics of the piglet gut microbiome during the weaning transition in association with health and nutrition. J. Anim. Sci. Biotechnol. 9, 1–9. doi: 10.1186/s40104-018-0269-6 30069307 PMC6065057

[ref47] Hamer H. M. Jonkers D. Venema K. Vanhoutvin S. Troost F. J. Brummer R.-J. (2007). Review article: the role of butyrate on colonic function. Aliment. Pharmacol. Ther. 27, 104–119. doi: 10.1111/j.1365-2036.2007.03562.x 17973645

[ref48] Havelaar A. H. Kirk M. D. Torgerson P. R. Gibb H. J. Hald T. Lake R. J.. (2015). World Health Organization global estimates and regional comparisons of the burden of foodborne disease in 2010. PLoS Med. 12:e1001923. doi: 10.1371/journal.pmed.1001923 26633896 PMC4668832

[ref49] Hays R. D. Morales L. S. (2001). The RAND-36 measure of health-related quality of life. Ann. Med. 33, 350–357. doi: 10.3109/07853890109002089 11491194

[ref50] Heinritz S. N. Mosenthin R. Weiss E. (2013). Use of pigs as a potential model for research into dietary modulation of the human gut microbiota. Nutr. Res. Rev. 26, 191–209. doi: 10.1017/S0954422413000152 24134811

[ref51] Heinritz S. N. Weiss E. Eklund M. Aumiller T. Louis S. Rings A.. (2016). Intestinal microbiota and microbial metabolites are changed in a pig model fed a high-fat/low-fiber or a low-fat/high-fiber diet. PLoS One 11, 1–21. doi: 10.1371/journal.pone.0154329 PMC4839692 27100182

[ref52] Hicks T. A. McGlone J. J. Whisnant C. S. Kattesh H. G. Norman R. L. (1998). Behavioral, endocrine, immune, and performance measures for pigs exposed to acute stress. J. Anim. Sci. 76, 474–483. doi: 10.2527/1998.762474x 9498355

[ref53] Hiippala K. Kainulainen V. Kalliomäki M. Arkkila P. Satokari R. (2016). Mucosal prevalence and interactions with the epithelium indicate commensalism of *Sutterella* spp. Front. Microbiol. 7:1706. doi: 10.3389/fmicb.2016.01706 27833600 PMC5080374

[ref54] Hoffman J. M. Margolis K. G. (2020). Building community in the gut: a role for mucosal serotonin. Nat. Rev. Gastroenterol. Hepatol. 17, 6–8. doi: 10.1038/s41575-019-0227-6 31624372 PMC6930332

[ref55] Holman D. B. Brunelle B. W. Trachsel J. Allen H. K. (2017). Meta-analysis to define a core microbiota in the swine gut. mSystems 2, e00004–e00017. doi: 10.1128/mSystems.00004-17 28567446 PMC5443231

[ref56] Holmstrøm K. Collins M. D. Møller T. Falsen E. Lawson P. A. (2004). *Subdoligranulum variabile* gen. nov., sp. nov. from human feces. Anaerobe 10, 197–203. doi: 10.1016/j.anaerobe.2004.01.004 16701519

[ref57] Hua X. Zhu J. Yang T. Guo M. Li Q. Chen J.. (2020). The gut microbiota and associated metabolites are altered in sleep disorder of children with autism spectrum disorders. Front. Psych. 11:855. doi: 10.3389/fpsyt.2020.00855 PMC7493623 32982808

[ref58] Huang Y. Shi X. Li Z. Shen Y. Shi X. Wang L.. (2018). Possible association of firmicutes in the gut microbiota of patients with major depressive disorder. Neuropsychiatr. Dis. Treat. 14, 3329–3337. doi: 10.2147/NDT.S188340 30584306 PMC6284853

[ref59] Hyun Y. Ellis M. Johnson R. W. (1998). Effects of feeder type, space allowance, and mixing on the growth performance and feed intake pattern of growing pigs. J. Anim. Sci. 76, 2771–2778. doi: 10.2527/1998.76112771x 9856385

[ref60] Ison S. H. D’Eath R. B. Robson S. K. Baxter E. M. Ormandy E. Douglas A. J.. (2010). “Subordination style” in pigs? The response of pregnant sows to mixing stress affects their offspring’s behaviour and stress reactivity. Appl. Anim. Behav. Sci. 124, 16–27. doi: 10.1016/j.applanim.2010.02.001

[ref61] Jackson M. A. Verdi S. Maxan M. E. Shin C. M. Zierer J. Bowyer R. C. E.. (2018). Gut microbiota associations with common diseases and prescription medications in a population-based cohort. Nat. Commun. 9, 1–8. doi: 10.1038/s41467-018-05184-7 29985401 PMC6037668

[ref62] Jarvis S. Moinard C. Robson S. K. Baxter E. Ormandy E. Douglas A. J.. (2006). Programming the offspring of the pig by prenatal social stress: Neuroendocrine activity and behaviour. Horm. Behav. 49, 68–80. doi: 10.1016/j.yhbeh.2005.05.004 15961089

[ref63] Jiang H. Ling Z. Zhang Y. Mao H. Ma Z. Yin Y.. (2015). Altered fecal microbiota composition in patients with major depressive disorder. Brain Behav. Immun. 48, 186–194. doi: 10.1016/j.bbi.2015.03.016 25882912

[ref64] Jin M. Li J. Liu F. Lyu N. Wang K. Wang L.. (2019). Analysis of the gut microflora in patients with Parkinson’s disease. Front. Neurosci. 13, 1–9. doi: 10.3389/fnins.2019.01184 31824239 PMC6883725

[ref65] Kelly J. R. Borre Y. OBrien C. Patterson E. El Aidy S. Deane J.. (2016). Transferring the blues: Depression-associated gut microbiota induces neurobehavioural changes in the rat. J. Psychiatr. Res. 82, 109–118. doi: 10.1016/j.jpsychires.2016.07.019 27491067

[ref66] Kelly C. P. LaMont J. T. (1998). *Clostridium difficile* infection. Annu. Rev. Med. 49, 375–390. doi: 10.1146/annurev.med.49.1.375 9509270

[ref67] Kemeny M. E. (2003). The psychobiology of stress. Curr. Dir. Psychol. Sci. 12, 124–129. doi: 10.1111/1467-8721.01246

[ref9003] Khan I. Bai Y. Zha L. Ullah N. Ullah H. Hussain S. R.. (2021). Mechanism of the Gut Microbiota Colonization Resistance and Enteric Pathogen Infection. Front. Cell. Infect. Microbiol. 11:716299. doi: 10.3389/fcimb.2021.716299 35004340 PMC8733563

[ref68] Kindaichi T. Yamaoka S. Uehara R. Ozaki N. Ohashi A. Albertsen M.. (2016). Phylogenetic diversity and ecophysiology of Candidate phylum Saccharibacteria in activated sludge. FEMS Microbiol. Ecol. 92:fiw078. doi: 10.1093/femsec/fiw078 27090759

[ref69] Kleiman S. C. Bulik-Sullivan E. C. Glenny E. M. Zerwas S. C. Huh E. Y. Tsilimigras M. C. B.. (2017). The gut-brain axis in healthy females: lack of significant association between microbial composition and diversity with psychiatric measures. PLoS One 12:e0170208. doi: 10.1371/journal.pone.0170208 28103291 PMC5245801

[ref70] Kuo P. H. Chung Y. C. E. (2019). Moody microbiome: Challenges and chances. J. Formos. Med. Assoc. 118, S42–S54. doi: 10.1016/j.jfma.2018.09.004 30262220

[ref71] Kushkevych I. Dordević D. Kollár P. (2018). Analysis of physiological parameters of Desulfovibrio strains from individuals with colitis. Open Life Sci. 13, 481–488. doi: 10.1515/biol-2018-0057 33817117 PMC7874683

[ref72] Kushkevych I. Dordević D. Vítězová M. (2021). Possible synergy effect of hydrogen sulfide and acetate produced by sulfate-reducing bacteria on inflammatory bowel disease development. J. Adv. Res. 27, 71–78. doi: 10.1016/j.jare.2020.03.007 33318867 PMC7728581

[ref73] Kverka M. Zakostelska Z. Klimesova K. Sokol D. Hudcovic T. Hrncir T.. (2011). Oral administration of *Parabacteroides distasonis* antigens attenuates experimental murine colitis through modulation of immunity and microbiota composition. Clin. Exp. Immunol. 163, 250–259. doi: 10.1111/j.1365-2249.2010.04286.x 21087444 PMC3043316

[ref74] Lee S. A. Kong C. Adeola O. Kim B. G. (2016). Different coefficients and exponents for metabolic body weight in a model to estimate individual feed intake for growing-finishing pigs. Asian-Australasian J. Anim. Sci. 29, 1756–1760. doi: 10.5713/ajas.16.0420 PMC5088424 27608642

[ref75] Levine U. Y. Bearson S. M. D. Stanton T. B. (2012). *Mitsuokella jalaludinii* inhibits growth of *Salmonella enterica* serovar Typhimurium. Vet. Microbiol. 159, 115–122. doi: 10.1016/j.vetmic.2012.03.027 22503601

[ref76] Li Y. Guo Y. Wen Z. Jiang X. Ma X. Han X. (2018). Weaning Stress Perturbs Gut Microbiome and Its Metabolic Profile in Piglets. Sci. Rep. 8:18068. doi: 10.1038/s41598-018-33649-8 30584255 PMC6305375

[ref77] Lima J. Auffret M. D. Stewart R. D. Dewhurst R. J. Duthie C.-A. Snelling T. J.. (2019). Identification of rumen microbial genes involved in pathways linked to appetite, growth, and feed conversion efficiency in cattle. Front. Genet. 10:701. doi: 10.3389/fgene.2019.00701 31440274 PMC6694183

[ref78] Lindemann M. D. Kim B. G. (2007). Technical note: A model to estimate individual feed intake of swine in group feeding. J. Anim. Sci. 85, 972–975. doi: 10.2527/jas.2006-412 17121980

[ref79] Liu Y. Zhang L. Wang X. Wang Z. Zhang J. Jiang R.. (2016). Similar fecal microbiota signatures in patients with diarrhea-predominant irritable bowel syndrome and patients with depression. Clin. Gastroenterol. Hepatol. 14, 1602–1611.e5. doi: 10.1016/j.cgh.2016.05.033 27266978

[ref80] Loubinoux J. Bronowicki J. P. Pereira I. A. C. Mougenel J. L. Le Faou A. E. (2002). Sulfate-reducing bacteria in human feces and their association with inflammatory bowel diseases. FEMS Microbiol. Ecol. 40, 107–112. doi: 10.1016/S0168-6496(02)00201-5 19709217

[ref81] Martin C. R. Osadchiy V. Kalani A. Mayer E. A. (2018). The brain-gut-microbiome axis. Cmgh 6, 133–148. doi: 10.1016/j.jcmgh.2018.04.003 30023410 PMC6047317

[ref82] Martínez-Miró S. Tecles F. Ramón M. Escribano D. Hernández F. Madrid J.. (2016). Causes, consequences and biomarkers of stress in swine: an update. BMC Vet. Res. 12:171. doi: 10.1186/s12917-016-0791-8 27543093 PMC4992232

[ref83] McGlone J. J. Salak J. L. Lumpkin E. A. Nicholson R. I. Gibson M. Norman R. L. (1993). Shipping stress and social status effects on pig performance, plasma cortisol, natural killer cell activity, and leukocyte numbers. J. Anim. Sci. 71, 888–896. doi: 10.2527/1993.714888x 8478291

[ref84] Messaoudi M. Lalonde R. Violle N. Javelot H. Desor D. Nejdi A.. (2011). Assessment of psychotropic-like properties of a probiotic formulation (*Lactobacillus helveticus* R0052 and *Bifidobacterium longum* R0175) in rats and human subjects. Br. J. Nutr. 105, 755–764. doi: 10.1017/S0007114510004319 20974015

[ref85] Mi J. Peng H. Wu Y. Wang Y. Liao X. (2019). Diversity and community of methanogens in the large intestine of finishing pigs. BMC Microbiol. 19:83. doi: 10.1186/s12866-019-1459-x 31035941 PMC6489232

[ref86] Michels N. Van de Wiele T. Fouhy F. O’Mahony S. Clarke G. Keane J. (2019). Gut microbiome patterns depending on children’s psychosocial stress: Reports versus biomarkers. Brain Behav. Immun. 80, 751–762. doi: 10.1016/j.bbi.2019.05.024 31112792

[ref87] Miller E. R. Ullrey D. E. (1987). The pig as a model for human nutrition. Annu. Rev. Nutr. 7, 361–382. doi: 10.1146/annurev.nu.07.070187.002045 3300739

[ref88] Misiak B. Łoniewski I. Marlicz W. Frydecka D. Szulc A. Rudzki L.. (2020). The HPA axis dysregulation in severe mental illness: Can we shift the blame to gut microbiota? Prog. Neuro-Psychopharmacol. Biol. Psychiatry 102:109951. doi: 10.1016/j.pnpbp.2020.109951 32335265

[ref89] Miyazaki M. Sakai S. Ritalahti K. M. Saito Y. Yamanaka Y. Saito Y.. (2014). Sphaerochaeta multiformis sp. nov., an anaerobic, psychrophilic bacterium isolated from subseafloor sediment, and emended description of the genus Sphaerochaeta. Int. J. Syst. Evol. Microbiol. 64, 4147–4154. doi: 10.1099/ijs.0.068148-0 25249566

[ref90] Mutua J. Y. Marshall K. Paul B. K. Notenbaert A. M. O. (2020). A methodology for mapping current and future heat stress risk in pigs. Animal 14, 1952–1960. doi: 10.1017/S1751731120000865 32349852 PMC7435152

[ref91] Oksanen J. Blanchet F. G. Friendly M. Kindt R. Legendre P. McGlinn D.. (2020). vegan: community ecology package. R package version 2, 5–7. Available at: https://cran.r-project.org/package=vegan

[ref92] Oyedele O. F. Lubbe S. (2015). The construction of a partial least-squares biplot. J. Appl. Stat. 42, 2449–2460. doi: 10.1080/02664763.2015.1043858

[ref93] Parada Venegas D. De la Fuente M. K. Landskron G. González M. J. Quera R. Dijkstra G.. (2019). Short chain fatty acids (SCFAs)-mediated gut epithelial and immune regulation and its relevance for inflammatory bowel diseases. Front. Immunol. 10:277. doi: 10.3389/fimmu.2019.00277 30915065 PMC6421268

[ref94] Patience J. F. Umboh J. F. Chaplin R. K. Nyachoti C. M. (2005). Nutritional and physiological responses of growing pigs exposed to a diurnal pattern of heat stress. Livest. Prod. Sci. 96, 205–214. doi: 10.1016/j.livprodsci.2005.01.012

[ref95] Prunier A. Mounier A. M. Hay M. (2005). Effects of castration, tooth resection, or tail docking on plasma metabolites and stress hormones in young pigs. J. Anim. Sci. 83, 216–222. doi: 10.2527/2005.831216x 15583062

[ref96] Randolph J. H. Cromwell G. L. Stahly T. S. Kratzer D. D. (1981). Effects of group size and space allowance on performance and behavior of swine. J. Anim. Sci. 53, 922–927. doi: 10.2527/jas1981.534922x

[ref97] Ritalahti K. M. Justicia-Leon S. D. Cusick K. D. Ramos-Hernandez N. Rubin M. Dornbush J.. (2011). *Sphaerochaeta globosa* gen. nov., sp. nov. and *sphaerochaeta pleomorpha* sp. nov., free-living, spherical spirochaetes. Int. J. Syst. Evol. Microbiol. 62, 210–216. doi: 10.1099/ijs.0.023986-0 21398503

[ref98] Roesch L. F. W. Dobbler P. T. Pylro V. S. Kolaczkowski B. Drew J. C. Triplett E. W. (2020). pime: A package for discovery of novel differences among microbial communities. Mol. Ecol. Resour. 20, 415–428. doi: 10.1111/1755-0998.13116 31698527

[ref99] Rohart F. Gautier B. Singh A. Lê Cao K.-A. (2017). mixOmics: An R package for ‘omics feature selection and multiple data integration. PLoS Comput. Biol. 13:e1005752. doi: 10.1371/journal.pcbi.1005752 29099853 PMC5687754

[ref100] Rose E. C. Blikslager A. T. Ziegler A. L. (2022). Porcine models of the intestinal microbiota: the translational key to understanding how gut commensals contribute to gastrointestinal disease. Front. Vet. Sci. 9, 1–8. doi: 10.3389/fvets.2022.834598 PMC8990160 35400098

[ref101] Rosero J. A. Killer J. Sechovcová H. Mrázek J. Benada O. Fliegerová K.. (2016). Reclassification of *Eubacterium rectale* (Hauduroy et al. 1937) prévot 1938 in a new genus agathobacter gen. nov. as Agathobacter rectalis comb. nov., and description of Agathobacter ruminis sp. nov., isolated from the rumen contents of sheep and cows. Int. J. Syst. Evol. Microbiol. 66, 768–773. doi: 10.1099/ijsem.0.000788 26619944

[ref102] Roura E. Koopmans S. J. Lallès J. P. Le Huerou-Luron I. De Jager N. Schuurman T.. (2016). Critical review evaluating the pig as a model for human nutritional physiology. Nutr. Res. Rev. 29, 60–90. doi: 10.1017/S0954422416000020 27176552

[ref103] Ruis M. A. W. Te Brake J. H. A. Engel B. Ekkel E. D. Buist W. G. Blokhuis H. J.. (1997). The circadian rhythm of salivary cortisol in growing pigs: Effects of age, gender, and stress. Physiol. Behav. 62, 623–630. doi: 10.1016/S0031-9384(97)00177-7 9272674

[ref104] Rutherford K. M. D. Haskell M. J. Glasbey C. Lawrence A. B. (2006). The responses of growing pigs to a chronic-intermittent stress treatment. Physiol. Behav. 89, 670–680. doi: 10.1016/j.physbeh.2006.08.006 16982073

[ref105] Scollo A. Gottardo F. Contiero B. Edwards S. A. (2014). Does stocking density modify affective state in pigs as assessed by cognitive bias, behavioural and physiological parameters? Appl. Anim. Behav. Sci. 153, 26–35. doi: 10.1016/j.applanim.2014.01.006

[ref106] Shetty S. A. Marathe N. P. Lanjekar V. Ranade D. Shouche Y. S. (2013). Comparative genome analysis of *Megasphaera* sp. reveals niche specialization and its potential role in the human gut. PLoS One 8:e79353. doi: 10.1371/journal.pone.0079353 24260205 PMC3832451

[ref107] Simpson C. A. Diaz-Arteche C. Eliby D. Schwartz O. S. Simmons J. G. Cowan C. S. M. (2021). The gut microbiota in anxiety and depression – A systematic review. Clin. Psychol. Rev. 83:101943. doi: 10.1016/j.cpr.2020.101943 33271426

[ref108] Smith S. M. Vale W. W. (2006). The role of the hypothalamic-pituitary-adrenal axis in neuroendocrine responses to stress. Dialogues Clin. Neurosci. 8, 383–395. doi: 10.31887/dcns.2006.8.4/ssmith 17290797 PMC3181830

[ref109] Songer J. G. Uzal F. A. (2005). Clostridial enteric infections in pigs. J. Vet. Diagnostic Investig. 17, 528–536. doi: 10.1177/104063870501700602 16475510

[ref110] Stewart D. Romo J. A. Lamendella R. Kumamoto C. A. (2019). The role of fungi in *C. difficile* infection: An underappreciated transkingdom interaction. Fungal Genet. Biol. 129, 1–6. doi: 10.1016/j.fgb.2019.04.007 30978391 PMC6642687

[ref111] Sun L. Zhang H. Cao Y. Wang C. Zhao C. Wang H.. (2019). Fluoxetine ameliorates dysbiosis in a depression model induced by chronic unpredicted mild stress in mice. Int. J. Med. Sci. 16, 1260–1270. doi: 10.7150/ijms.37322 31588192 PMC6775263

[ref112] Takada T. Kurakawa T. Tsuji H. Nomoto K. (2019). “Fusicatenibacter. In: Bergeys Manual of Systematics of Archaea and Bacteria (Wiley), 1–5. Available at: https://onlinelibrary.wiley.com/doi/10.1002/9781118960608.gbm01639.

[ref113] Tsairidou S. Anacleto O. Woolliams J. A. Doeschl-Wilson A. (2019). Enhancing genetic disease control by selecting for lower host infectivity and susceptibility. Heredity (Edinb). 122, 742–758. doi: 10.1038/s41437-018-0176-9 30651590 PMC6781107

[ref114] Tsinganou E. Gebbers J.-O. (2010). Human intestinal spirochetosis-a review. Ger. Med. Sci. 8, 1–7. doi: 10.3205/000090 PMC2830567 20200654

[ref115] Tsukahara T. Hashizume K. Koyama H. Ushida K. (2006). Stimulation of butyrate production through the metabolic interaction among lactic acid bacteria, Lactobacillus acidophilus, and lactic acid-utilizing bacteria, *Megasphaera elsdenii*, in porcine cecal digesta. Anim. Sci. J. 77, 454–461. doi: 10.1111/j.1740-0929.2006.00372.x

[ref116] Turner S. P. Horgan G. W. Edwards S. A. (2001). Effect of social group size on aggressive behaviour between unacquainted domestic pigs. Appl. Anim. Behav. Sci. 74, 203–215. doi: 10.1016/S0168-1591(01)00168-X

[ref117] Turner S. P. White I. M. S. Brotherstone S. Farnworth M. J. Knap P. W. Penny P.. (2006). Heritability of post-mixing aggressiveness in grower-stage pigs and its relationship with production traits. Anim. Sci. 82, 615–620. doi: 10.1079/ASC200678

[ref118] Valles-Colomer M. Falony G. Darzi Y. Tigchelaar E. F. Wang J. Tito R. Y.. (2019). The neuroactive potential of the human gut microbiota in quality of life and depression. Nat. Microbiol. 4, 623–632. doi: 10.1038/s41564-018-0337-x 30718848

[ref119] van der Beek C. M. Dejong C. H. C. Troost F. J. Masclee A. A. M. Lenaerts K. (2017). Role of short-chain fatty acids in colonic inflammation, carcinogenesis, and mucosal protection and healing. Nutr. Rev. 75, 286–305. doi: 10.1093/nutrit/nuw067 28402523

[ref120] Veech J. A. (2017). “Measuring biodiversity,” In: *The Encyclopedia of the Anthropocene*, eds. DellaSala D. A. Goldstein M. I. (Oxford, UK: Elsevier Inc.), 287–295.

[ref121] Wang Y. Kasper L. H. (2014). The role of microbiome in central nervous system disorders. Brain Behav. Immun. 38, 1–12. doi: 10.1016/j.bbi.2013.12.015 24370461 PMC4062078

[ref122] Warton D. I. Wright S. T. Wang Y. (2012). Distance-based multivariate analyses confound location and dispersion effects. Methods Ecol. Evol. 3, 89–101. doi: 10.1111/j.2041-210X.2011.00127.x

[ref123] West J. W. (2003). Effects of heat-stress on production in dairy cattle. J. Dairy Sci. 86, 2131–2144. doi: 10.3168/jds.S0022-0302(03)73803-X 12836950

[ref124] Yu Z. Morrison M. (2004). Improved extraction of PCR-quality community DNA from digesta and fecal samples. BioTechniques 36, 808–812. doi: 10.2144/04365st04 15152600

[ref125] Zaboli G. Huang X. Feng X. Ahn D. U. (2019). How can heat stress affect chicken meat quality? - A review. Poult. Sci. 98, 1551–1556. doi: 10.3382/ps/pey399 30169735

[ref126] Ziegler M. G. (2012). “Psychological stress and the autonomic nervous system” in Primer on the Autonomic Nervous System. (San Diego, USA: Academic Press), 291–293.

