# Supplementary material for: Identification of intestinal and fecal microbial biomarkers using a porcine social stress model
Source: Front Microbiol. 2023 Nov 9;14:1197371. doi: 10.3389/fmicb.2023.1197371 (PMC10670831; doi:10.3389/fmicb.2023.1197371)
Supplement: Supplementary file 1 [file Data_Sheet_1.pdf]

Table S1 Summary information of the important genera (VIP>0.8) in the caecum, including mean relative abundance, mean abundance in clr form, their coefficient of variation, Variable Importance in Projection (VIP), the regression coefficient due to stress, the contrast between stress and control with clr data and p-value of Welsh's t-test by ANOVA-Like Differential Expression analysis.

	Descriptive statistics				DA-PLS approach (in clr)		Differences between treatment groups (Stress - Control) ALDEx clr data	
	Mean relative abundance (%)	Coef. Variation	Mean abundance (in clr)	Coef. Variation	VIP	Regression coefficient - Stress	Differences between groups	p.Welch
Anaerovibrio	3.0629	0.3388	2.5584	0.3052	1.233	-0.072	-0.8277	6.97E-05*
Parabacteroides	0.1928	1.8074	-4.7326	-0.8219	1.164	0.068	4.4555	4.38E-03*
Megasphaera	2.1087	0.4760	1.9077	0.6190	1.141	-0.066	-1.4305	2.62E-04*
Faecalibacterium	6.1326	0.3322	3.5523	0.2532	1.14	-0.066	-0.8746	3.81E-04*
Dialister	3.6165	0.5058	2.6409	0.4888	1.11	-0.064	-1.3799	4.72E-04*
Turicibacter	0.2195	1.2641	-2.3173	-0.8936	1.058	0.061	1.6797	9.22E-03*
Romboutsia	0.2064	1.1057	-2.3292	-0.9121	1.021	0.059	1.5849	1.27E-02*
Prevotella	37.8692	0.1308	6.2647	0.0899	1.012	-0.059	-0.5334	1.15E-03*
Catenibacterium	2.0946	1.0943	1.4034	1.3742	0.994	-0.058	-1.7377	2.03E-03*
Solobacterium	1.4653	0.3423	1.5140	0.3632	0.97	-0.056	-0.5153	2.19E-03*
Oribacterium	0.9967	0.3177	0.9678	0.7188	0.961	-0.056	-0.6380	2.11E-03*
Clostridium	3.0433	0.7091	2.2193	0.5057	0.875	0.051	0.9937	2.55E-02*
Fusicatenibacter	0.3808	0.3488	-0.4463	-1.8046	0.84	-0.049	-0.5818	7.60E-03*
Subdoligranulum	3.8129	0.3241	2.8915	0.1793	0.812	-0.047	-0.4018	6.92E-03*
Terrisporobacter	1.9658	0.6550	1.6555	0.5694	0.766	0.044	0.7568	6.34E-02*
Agathobacter	1.8571	0.4085	1.8282	0.3817	0.729	-0.042	-0.4043	1.61E-02*

Table S2 Summary information of the important genera (VIP>0.8) in the colon, including mean relative abundance, mean abundance in clr form, their coefficient of variation, Variable Importance in Projection (VIP), the regression coefficient due to stress, the contrast between stress and control with clr data and p-value of Welsh's t-test by ANOVA-Like Differential Expression analysis.

	Descriptive statistics				DA-PLS approach (in clr data)		Differences between treatment groups (Stress - Control) ALDEx clr data	
	Mean relative abundance (%)	Coef. Variation	Mean abundance (in clr)	Coef. Variation	VIP	Regression coefficient - Stress	Differences between groups	p.Welch
Anaerovibrio	2.518	0.3285	2.3400	0.3306	1.299	-0.054	-0.8420	7.79E-05*
Faecalibacterium	5.947	0.3354	3.5808	0.2572	1.233	-0.051	-0.9346	1.93E-04*
Coprococcus	1.039	0.2961	1.0860	0.5714	1.203	-0.05	-0.6649	3.26E-04*
Megasphaera	2.276	0.4198	2.1246	0.4974	1.106	-0.046	-1.0873	4.87E-04*
Dialister	3.698	0.4384	2.8371	0.3564	1.091	-0.045	-1.0575	8.69E-04*
Catenibacterium	2.353	0.8361	1.8308	0.8953	1.027	-0.043	-1.3730	1.60E-03*
Prevotella	35.951	0.1274	6.2510	0.0846	1.025	-0.043	-0.4937	1.23E-03*
Fusicatenibacter	0.432	0.3797	-0.2285	-4.1053	1.013	-0.042	-0.7348	1.21E-03*
Alloprevotella	6.177	0.3150	3.6512	0.1703	1.008	-0.042	-0.5260	1.14E-03*
Parabacteroides	0.243	1.0758	-3.0039	-1.1520	0.996	0.041	2.1275	1.84E-02*
Agathobacter	1.687	0.4716	1.7207	0.5208	0.990	-0.041	-0.8806	2.26E-03*
Allisonella	0.102	0.4968	-2.3748	-0.4453	0.955	-0.04	-0.9332	3.80E-03*
Solobacterium	1.567	0.3069	1.6760	0.3691	0.931	-0.039	-0.5055	2.51E-03*
Oribacterium	1.161	0.3201	1.2468	0.5592	0.916	-0.038	-0.4964	4.87E-03*
Holdemanella	1.449	0.3902	1.5420	0.4912	0.852	-0.035	-0.5895	2.67E-03*
Desulfovibrio	0.288	1.0205	-1.8980	-1.2212	0.849	0.035	1.4078	4.02E-02*
Clostridium	2.593	0.7335	2.0653	0.5207	0.845	0.035	0.8533	9.45E-02*
Treponema	0.616	1.5638	-0.8710	-2.2948	0.838	0.035	1.2600	5.20E-02*
Butyricicoccus	0.318	0.3597	-0.6393	-1.2592	0.816	-0.034	-0.4888	6.66E-03*
Sutterella	0.570	0.5748	0.0462	22.2835	0.816	-0.034	-0.7589	8.96E-03*

Table S3 Summary information of the important genera (VIP>0.8) in the faeces, including mean relative abundance, mean abundance in clr form, their coefficient of variation, Variable Importance in Projection (VIP), the regression coefficient due to stress, the contrast between stress and control with clr data and p-value of Welsh's t-test by ANOVA-Like Differential Expression analysis.

	Descriptive statistics				DA-PLS approach (in clr data)		Differences between treatment groups (Stress - Control) ALDEx clr data	
	Mean relative abundance (%)	Coef. Variation	Mean abundance (in clr)	Coef. Variation	VIP	Regression coefficient - Stress	Differences between groups	p.Welch
Clostridium	4.2281	1.1203	1.4839	1.7899	1.367	0.046	2.7706	8.52E-04*
Dialister	2.4688	0.5833	1.8835	0.6355	1.317	-0.044	-1.0151	2.68E-04*
Anaerovibrio	1.9117	0.5724	1.5171	0.7689	1.317	-0.044	-1.1338	1.90E-04*
Faecalibacterium	2.9572	0.6369	2.1145	0.5435	1.270	-0.043	-1.1501	6.19E-04*
Parabacteroides	0.4779	0.6279	-0.5622	-2.0244	1.177	0.039	0.9466	4.55E-03*
Methanobrevibacter	1.1510	1.2624	-0.6788	-4.4837	1.110	0.037	2.3139	7.15E-03*
Mitsuokella	0.7979	1.0130	0.0211	67.1812	1.038	-0.035	-0.9833	5.93E-03*
Holdemanella	1.1578	0.4695	0.9222	0.8759	1.026	-0.034	-0.6197	5.79E-03*
Sphaerochaeta	1.0282	0.7118	0.4707	2.5423	1.022	0.034	1.0227	1.36E-02*
Terrisporobacter	2.1592	0.8548	1.1051	1.8781	0.979	0.033	1.4102	2.34E-02*
Catenibacterium	2.4788	0.7299	1.4547	1.5595	0.964	-0.032	-1.2872	1.48E-02*
Methanosphaera	0.4246	1.0585	-1.3429	-1.4624	0.963	0.032	1.4184	2.85E-02*
Candidatus.Saccharimonas	0.2160	1.0951	-2.3549	-0.8749	0.910	0.03	1.2045	4.23E-02*
Prevotella	29.8997	0.2566	5.7191	0.1159	0.866	-0.029	-0.3940	1.31E-02*
Butyricicoccus	0.1967	0.6386	-2.2398	-1.0482	0.865	-0.029	-1.2405	6.18E-02 ^{ns}
Libanicoccus	0.2006	1.0356	-2.7678	-1.0314	0.862	-0.029	-1.5195	4.17E-02*
Fusicatenibacter	0.5107	0.4505	-0.4260	-3.7989	0.860	-0.029	-0.7190	2.92E-02*
Marvinbryantia	0.2739	0.7226	-1.9721	-1.2908	0.858	0.029	0.6574	5.66E-02 ^{ns}
Oribacterium	1.3237	0.4338	1.1419	0.7340	0.848	-0.028	-0.5527	1.44E-02*
Alloprevotella	2.9859	0.3721	2.3284	0.3231	0.831	-0.028	-0.4479	1.50E-02*
Desulfovibrio	0.7021	0.5812	0.1186	7.2389	0.803	0.027	0.5811	9.73E-02 ^{ns}
Agathobacter	0.8789	0.8302	-1.1269	-3.6018	0.801	-0.027	-1.6542	6.10E-02 ^{ns}
Streptococcus	0.9547	1.1141	-0.6835	-3.9833	0.785	0.026	1.4303	7.57E-02 ^{ns}
Treponema	5.5226	1.2228	2.2215	0.8263	0.763	0.026	0.8397	7.36E-02 ^{ns}
