# Supplementary material for: Identification of intestinal and fecal microbial biomarkers using a porcine social stress model
Source: Front Microbiol. 2023 Nov 9;14:1197371. doi: 10.3389/fmicb.2023.1197371 (PMC10670831; doi:10.3389/fmicb.2023.1197371)
Supplement: Supplementary file 2 [file Data_Sheet_2.pdf]

Table S4. Mean and the difference between treatment and resistant status of four pathogenic genera clr-transformed abundances

		Treatment				Resistant status			
		Stress (SE)	Control (SE)	Contrast (95HPD)	Pr0	Susceptible (SE)	Resistant (SE)	Contrast (95HPD)	Pr0
Caecum	*Campylobacter*	-1.75 (0.48)	-1.92 (0.43)	0.17 (-0.90 1.46)	0.69	0.02 (0.61)	-3.69 (0.38)	3.71 (2.36 4.87)	1.00
	Clostridium	3.45 (0.44)	2.34 (0.37)	1.11 (0.45 1.85)	1.00	3.66 (0.72)	2.13 (0.17)	1.52 (-0.11 2.97)	0.96
	Streptococcus	1.06 (0.41)	0.53 (0.36)	0.54 (-0.57 1.42)	0.80	2.09 (0.54)	-0.49 (0.26)	2.58 (1.30 3.86)	1.00
	Treponema	-0.73 (0.54)	-2.26 (0.52)	1.53 (0.05 3.06)	0.98	0.39 (0.69)	-3.38 (0.40)	3.77 (2.07 5.43)	1.00
Colon	*Campylobacter*	-0.34 (0.30)	-0.93 (0.28)	0.59 (-0.15 1.42)	0.95	0.63 (0.37)	-1.91 (0.22)	2.54 (1.66 3.48)	1.00
	Clostridium	3.89 (0.51)	2.94 (0.47)	0.95 (0.37 1.63)	1.00	4.84 (0.92)	1.99 (0.15)	2.85 (0.72 4.78)	1.00
	Streptococcus	0.97 (0.57)	0.80 (0.57)	0.17 (-1.29 1.41)	0.54	2.06 (0.85)	-0.29 (0.37)	2.35 (0.35 4.17)	0.99
	Treponema	0.45 (0.38)	-1.04 (0.38)	1.48 (0.60 2.42)	1.00	0.92 (0.49)	-1.51 (0.29)	2.43 (0.86 3.22)	1.00
Faeces	*Campylobacter*	-0.17 (0.23)	-0.29 (0.28)	0.12 (-0.45 0.71)	0.69	0.74 (0.40)	-1.21 (0.15)	1.95 (1.04 2.96)	1.00
	Clostridium	4.15 (0.57)	0.87 (0.51)	3.28 (1.96 4.47)	1.00	4.01 (0.80)	1.01 (0.35)	3.00 (1.21 4.73)	1.00
	Streptococcus	1.53 (0.54)	-0.83 (0.49)	2.36 (0.95 3.19)	1.00	2.31 (0.68)	-1.61 (0.38)	3.93 (2.86 5.24)	1.00
	Treponema	3.05 (0.19)	2.43 (0.20)	0.62 (0.07 1.19)	1.00	4.38 (0.23)	1.01 (0.16)	3.28 (2.69 3.86)	1.00

SE, standard error, 95HPD, 95% highest posterior density interval and Pr0, posterior probability of being positive
