# Supplementary material for: Identification of intestinal and fecal microbial biomarkers using a porcine social stress model
Source: Front Microbiol. 2023 Nov 9;14:1197371. doi: 10.3389/fmicb.2023.1197371 (PMC10670831; doi:10.3389/fmicb.2023.1197371)
Supplement: Supplementary file 3 [file Data_Sheet_3.pdf]

Pig ID	Farm Group	Litter	Pen Week4	Treatment	Skin lesion Week4	Cortisol AUC Week4
C1	2	BF410	C1	CONTROL	32	122.27
C2	1	BF457	C1	CONTROL	39	171.38
C3	3	GF4	C1	CONTROL	23	291.18
C4	2	BF437	C2	CONTROL	54	168.36
C5	2	BF460	C2	CONTROL	26	148.68
C6	3	3270	C2	CONTROL	101	300.06
C7	1	BF428	C2	CONTROL	47	184.22
C8	2	BF410	C3	CONTROL	94	142.37
C9	3	GF2	C3	CONTROL	25	89.49
C10	1	BF457	C3	CONTROL	75	38.97
C11	3	GF4	C3	CONTROL	21	91.03
C12	1	3279	C4	CONTROL	53	210.54
C13	2	BF437	C4	CONTROL	35	169.96
C14	2	BF410	C4	CONTROL	47	165.62
C15	3	GF2	C4	CONTROL	46	334.23
C16	1	3279	C5	CONTROL	16	72.22
C17	2	BF437	C5	CONTROL	39	117.73
C18	2	BF410	C5	CONTROL	44	208.11
C19	3	GF2	C5	CONTROL	38	208.41
S1	2	BF460	S10	STRESS	115	276.12
S2	1	BF457	S7	STRESS	69	257.57
S3	2	BF410	S6	STRESS	185	186.46
S4	2	BF460	S8	STRESS	122	365.05
S5	1	BF428	S9	STRESS	107	765.59
S6	1	3279	S6	STRESS	133	314.52
S7	2	BF460	S8	STRESS	106	267.39
S8	3	3270	S7	STRESS	126	398.44
S9	1	BF457	S8	STRESS	68	155.32
S10	2	BF410	S10	STRESS	150	317.28
S11	2	BF437	S7	STRESS	126	559.47
S12	3	GF2	S9	STRESS	131	528.2
S13	3	GF4	S9	STRESS	195	166.45
S14	1	BF428	S6	STRESS	25	217.65
S15	1	BF457	S10	STRESS	125	618.62
S16	3	3270	S6	STRESS	149	476.51
S17	3	3270	S10	STRESS	146	271.59
S18	1	BF457	S7	STRESS	251	582.89
S19	1	BF428	S9	STRESS	79	138.79
